# Carbon Binder Domain Inhomogeneity in Silicon‐Monoxide/Graphite Composite Anode by 2D Multiphysics Modeling

**DOI:** 10.1002/advs.202400729

**Published:** 2024-05-22

**Authors:** Xiang Gao, Jun Xu

**Affiliations:** ^1^ Department of Mechanical Engineering University of Delaware Newark DE 19716 USA; ^2^ Energy Mechanics and Sustainability Laboratory (EMSLab) University of Delaware Newark DE 19716 USA

**Keywords:** CBD inhomogeneity, high‐energy‐density battery, multiphysics modeling, SiO/graphite composite anode

## Abstract

The Carbon‐binder domain (CBD) plays a pivotal role in the performance of lithium‐ion battery electrodes. The heterogeneous distribution of CBD across the electrode has garnered significant attention. However, a thorough understanding of how this CBD inhomogeneity affects anode performance remains a crucial pursuit, especially when considering the inherent material variations present in the SiO/Graphite (SiO/Gr) composite anode. In this study, an electro‐chemo‐mechanical model is established that provides a detailed geometric description of the particles. This model allows to quantitatively uncover the effects of CBD inhomogeneity on the fundamental behaviors of the SiO/Gr composite anode. The findings indicate that reducing the proportion of CBD in the upper domain (near the anode surface) compared to the lower domain (near the current collector) positively influences electrochemical performance, particularly in terms of capacity and Li plating. However, such an arrangement introduces potential risks of mechanical failures, and it is recommended to incorporate a higher proportion of CBD alongside the SiO particles. Finally, an anode design with a lower CBD proportion in the upper domain exhibits superior rate performance. This study represents a pioneering modeling exploration of CBD inhomogeneity, offering a promising multiphysics model with significant potential for informing advanced battery design considerations.

## Graphical abstract

An electrochemo‐mechanical model is built to explore the impact of carbon‐binder domain (CBD) inhomogeneity on SiO/Graphite composite anode performance. It reveals that a lower CBD proportion near the anode surface enhances electrochemical performance but may increase mechanical failure risks. Optimizing CBD distribution, especially around SiO particles, can lead to superior rate performance, marking a significant step in battery design.

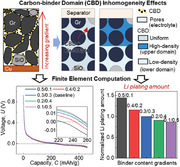

## 1 Introduction

**Figure 1:**
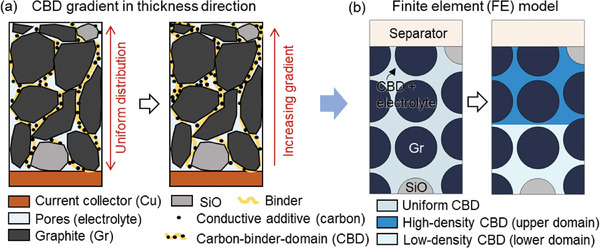
Illustration of a) the CBD distribution within the anode coating layers regarding the uniform distribution (left) and a CBD gradient (right); b) the finite element (FE) models representing the anode with uniform CBD distribution and CBD gradient, respectively.

Lithium‐ion batteries (LIBs) have emerged as a preeminent energy storage solution, finding widespread application in diverse sectors including electric vehicles (EVs),^[^ ^1^, ^2^ ^]^ portable electronics,^[^ ^3^, ^4^ ^]^ aviation,^[^ ^5^, ^6^ ^]^ and others. This enduring popularity has engendered significant interest within the research community with the continuing focus on advancing LIB technology in crucial domains such as energy density,^[^ ^7^, ^8^ ^]^ cyclability and longevity/durability,^[^ ^9^, ^10^ ^]^ and safety.^[^ ^11^, ^12^, ^13^ ^]^ The structural design of electrodes has attracted significant attentions as a pivotal factor influencing the array of performances mentioned above.^[^ ^14^, ^15^, ^16^, ^17^ ^]^ In the fabrication of lithium‐ion battery (LIB) electrodes, the arrangement of active particles and the carbon‐binder domain (CBD) is generally attained post the coating‐drying‐calendaring sequence without much design,^[^ ^18^, ^19^, ^20^ ^]^ unless a bespoke manual design approach is implemented. Yet, within the drying process, the distribution of the CBD may inadvertently impact the gradient across the electrode thickness (**Figure** **1**) due to solvent evaporation.^[^ ^21^, ^22^, ^23^, ^24^, ^25^ ^]^ This implies that unintentional inhomogeneity can be introduced into the electrode composition. To comprehensively investigate the impact of such inhomogeneity, a multitude of researchers have undertaken extensive studies employing both experimental and numerical approaches.^[^ ^26^, ^27^, ^28^, ^29^ ^]^ Within this body of research, certain studies have concentrated on elucidating the mechanisms behind the formation of this gradient and the influential factors governing its development.^[^ ^30^, ^31^ ^]^ Available literature have explored the gradient effect on battery performance, with a primary emphasis placed on investigating the cathode side. For example, Yari et al.^[^ ^26^ ^]^ identified two distinct types of inhomogeneity within the porous LiNi_0.6_Mn_0.2_Co_0.2_O_2_ cathode. These categories were classified as “constructive”, characterized by a CBD proportion larger near the current collector than near the separator, and “destructive”, exhibiting the opposite distribution. A reduction of up to 20% in cell rate performance was observed, with the extent of decline contingent on the degree of inhomogeneity. To the best of our knowledge, no available literature has discussed the impact of CBD gradient effects on the anode side.

Furthermore, the growing demand for high energy density has prompted to explore high‐capacity anode materials, such as the Li metal anode,^[^ ^32^, ^33^, ^34^, ^35^ ^]^ Sn anode,^[^ ^36^, ^37^, ^38^ ^]^ and Si‐based (SiO and Ferro Si) anode^[^ ^16^, ^39^, ^40^, ^41^ ^]^ and among others. Among these emerging anode candidates, Si‐based materials stand out as particularly promising on a commercial scale due to their cost‐effectiveness,^[^ ^42^ ^]^ earth abundance,^[^ ^43^ ^]^ and substantial theoretical galvanic capacity.^[^ ^44^, ^45^, ^46^ ^]^ Notably, when combined with traditional graphite (Gr) materials, the overall performance is significantly enhanced by mitigating the volumetric changes induced by lithiation.^[^ ^47^, ^48^ ^]^ However, Si‐based/Gr composites introduce heightened complexity due to their inherent inhomogeneity, further compounded by the presence of CBD inhomogeneity. This intricate interplay necessitates a comprehensive understanding of the interrelation and joint effects between these two forms of inhomogeneities.

In the present study, an electro‐chemo‐mechanics coupled modeling framework for SiO/Gr composite anode materials is established following our previous modeling strategy.^[^ ^39^ ^]^ In that study, a comprehensive analysis of fundamental multiphysics, incorporating detailed particle geometries, was conducted. The discussion delved into the parametric effects of SiO proportion, SiO distribution, and SiO size. The findings indicate that an optimal configuration involves ≈7 wt.% SiO, positioning SiO near the separator, and utilizing small SiO particles. In this study, a fundamental and quantitative understanding of the CBD inhomogeneity effects (here, the term “inhomogeneity” refers to a simplified two‐layered structure characterized by two different CBD contents, see Experimental Section) on the SiO/Gr anode performance are provided based on the modeling results. This paper is organized as follows. First, typical electrochemical‐mechanical behaviors of the SiO/Gr composite anode considering CBD inhomogeneity are introduced. Then, the coupling effects of SiO locations and CBD inhomogeneities are discussed in terms of electrochemical performance and mechanical behaviors. The rate performance of the anode with various CBD inhomogeneities is also analyzed. Finally, the conclusions are summarized.

The multiphysics modeling methodology, the coupling strategy, and the parameter settings are introduced in the Experimental Section.

## 2 Results

The effects of CBD inhomogeneity on the cell performance are examined here as the typical results. The typical results here are the plots of the selected parameters for both electrochemical (voltage, component state of charge (SOC), Li concentration in both solid and liquid phases, and the Li plating overpotential and amount) and mechanical (force and deformation) fields. The CBD inhomogeneity is achieved by setting different CBD proportions in the two domains (Figure 1b). The CBD proportion mentioned in the following parts is determined as the CBD fraction in the CBD and electrolyte combined domain (CBD+E) (details can be found in Experimental Section). Here we considered the pure Gr anode as the reference. The detailed analysis of the typical results are as follows:

### 2.1 Electrochemical Behavior

**Figure 2:**
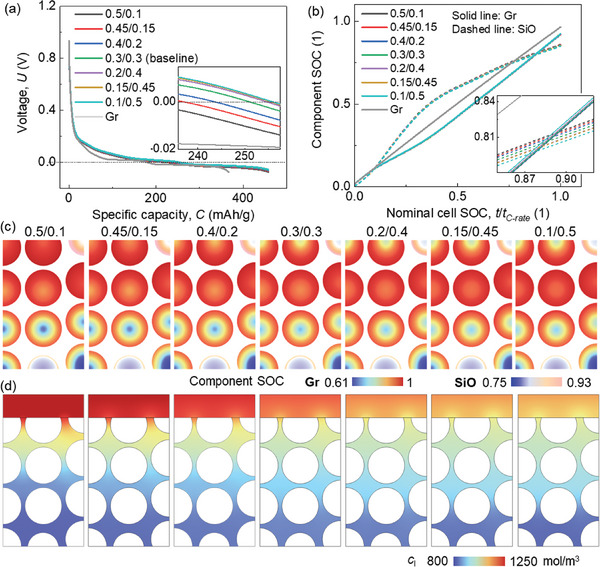
Computation results of the SiO/Gr composite anode (SiO ≈5%) with various CBD inhomogeneities during the lithiation process about a) voltage profile; b) component SOCs of SiO and Gr materials; c) the component SOCs and d) liquid phase concentration distributions at the end of lithiation.

For the electrochemical behavior, a slight difference is observed for the half‐cell voltage profiles (**Figure** **2**a). This difference is less sensitive to the CBD inhomogeneity in SiO/Gr anode than that in the pure Gr anode (maximum voltage variations between case‐0.5/0.1 and case‐0.4/0.2 are 0.006184 and 0.007387 V in SiO/Gr anode and Gr anode (Figure S1a, Supporting Information), respectively). According to the zoom‐in figure in Figure 2a, it is revealed that the voltage is higher when the upper domain processes small CBD proportion. This effect is more obvious in Case I to Case IV when the CBD proportion in the upper domain is still larger than that in the lower domain. The component SOC (*c*/*c*_max_ for the corresponding component Gr or SiO) curves demonstrate little difference among various CBD inhomogeneity cases (Figure 2b). It uncovers that the impact of CBD inhomogeneity is more pronounced in SiO compared to Gr. Specifically, a lower SOC in SiO is observed with a reduced CBD proportion in the upper domain. In the case of Gr particles, the CBD inhomogeneity demonstrates an opposite trend to that observed in SiO, yet with an extremely small magnitude. However, in the pure Gr anode, there is almost no difference of the active particle SOCs among all the CBD inhomogeneity cases (Figure S1b, Supporting Information).

When looking at the detailed distribution of the Li ions in both solid (Figure 2c; Video S1, Supporting Information) and liquid phases (Figure 2d), a clear correlation emerges between the polarization and the CBD inhomogeneity change. It indicates that reducing the CBD proportion in the surrounding area leads to an increased Li^+^ concentration gradient along the radial direction in both SiO and Gr particles. This phenomenon can primarily be attributed to the fact that the decrease in CBD proportion results in a relatively lower solid conductivity within the respective domain.^[^ ^49^, ^50^ ^]^ As a result, the solid current density flows are affected, subsequently influencing the electrochemical activity of the particles within that domain. Thus, transitioning from Case I to Case VII shows an increased gradient in the upper domain and a decreased gradient in the lower domain, which results in a more uniform distribution of Li^+^ (Figure 2c). Consequently, the gradient along the electrode thickness direction becomes smaller, which is consistent with the smaller polarization in the electrolyte (Figure 2d). Similar tendencies of the Li+ concentration distribution can be found in the pure Gr anode (Figure S1c,d, Supporting Information). This indicates that although the overall SOC is not affected by the CBD gradient, reducing the CBD proportion in the upper domain still benefits the Gr anode by leading to a more uniform distribution of Li^+^.

**Figure 3:**
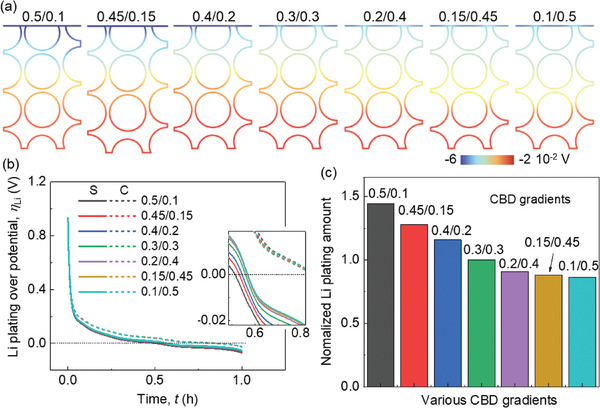
Computation results of the SiO/Gr composite anode (SiO ≈5%) with various CBD inhomogeneities during the lithiation process about a) Li plating overpotential distribution at the end of the lithiation process; b) the evolution of the Li plating overpotentials during the lithiation process at one point near the separator (S) and the other one near the current collector (C); c) the Li plating amount (normalized base on the baseline case (0.3/0.3).

The Li plating behaviors are discussed by analyzing the calculated Li plating overpotential behaviors. The basic Li plating criteria in our model is described in the Experimental Section. The overpotential for Li plating exhibits a linear gradient across the thickness where the values distributed near the separator side are more negative (**Figure** **3**a; Video S1, Supporting Information). It agrees with previous studies that the Li plating usually occurs near the separator sides first during lithiation. As the proportion of CBD in the upper domain decreases, the overpotential values near the separator become less negative, indicating an improved Li plating condition. Two specific points (i) at the separator‐anode interface, and ii) at the current collector‐anode interface) are selected to further study the evolution of the Li plating overpotential during the entire lithiation process (Figure 3b). However, notable distinctions emerge in the overpotentials near the separator, while less difference is observed for the overpotentials near the current collector. Consequently, employing the formula detailed in the Experimental Section, the Li plating quantities for each case were computed and compared (Figure 3c). In summary, reducing the CBD concentration in the upper domain compared to the lower domain proves advantageous in mitigating Li plating.

### 2.2 Mechanical Deformation Behavior with Various CBD Inhomogeneities

**Figure 4:**
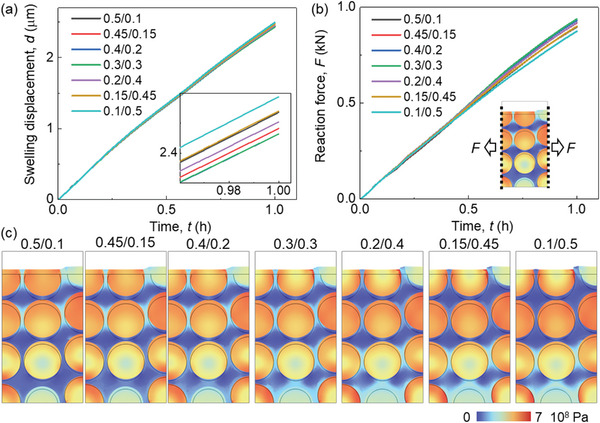
Comparison of mechanical behaviors among models with various CBD inhomogeneities of SiO/Gr anode (SiO w ≈ 5%) about the a) average thickness change; b) average swelling force in the in‐plane direction; c) Von Mises stress distribution and the deformation (the frame shows the original shape) at the end of the lithiation process (3600 s).

The CBD inhomogeneity has a small effect (<1%) on the lithiation induced swelling behavior of the electrode (**Figure** **4**a). The zoomed‐in picture in Figure 4a illustrates that the overall deformation initially decreases as the CBD proportion of the upper domain decreases. Subsequently, it begins to increase as the CBD proportion of the upper domain continues to decrease. Ultimately, the analysis unveils that Cases III (0.4/0.2) and IV (0.3/0.3) yield the most minimal deformation, whereas Case VII (0.1/0.5) demonstrates the most substantial deformation. This is the coupling result of particle swelling and CBD deformation. Based on the preceding discussion, it is evident that as the CBD proportions decrease within the upper domain, the Gr and SiO particles exhibit reduced lithiation, leading to a lesser extent of volume change. As the CBD proportion diminishes in the upper domain, the mechanical restraint imposed by the CBD on the particles correspondingly lessens. This reduction in constraint results in more pronounced deformation and greater mobility of the neighboring particles. Hence, the comprehensive deformation pattern exhibited by the entire electrode demonstrates a sequential trend of decreasing followed by increasing behavior as the CBD proportions decrease within the upper domain. The swelling forces exhibit a contrasting trend to the deformation behaviors: initially, they increase and subsequently decrease with a reduction in CBD proportion within the upper domain (Figure 4b). This can be directly analyzed by the deformation behavior that the large deformation of the electrode demonstrates the small overall constraint along the thickness direction, which further leads to the small swelling force along the in‐plane direction. Besides, the detailed stress distribution can also help explain this. The stress in Gr particles in the upper domain decreases due to the less lithiation, while the stress in the SiO increases, according to the opposite mechanical properties of Gr and SiO (see Experimental Section). Contrasting stress evolutions can also be observed within the lower domain, although they exhibit an inverse pattern when compared to the upper domain. Consequently, the overall stress evolution does not follow a monotonic trajectory in response to changes in CBD inhomogeneity. This in‐plane force signifies the potential for generating electrode curvature and causing the detachment of coating layers from the current collector. Consequently, Case VII exhibits the most favorable behavior in relation to this potential failure.

The intricate stress and deformation patterns within the electrodes, characterized by diverse CBD inhomogeneities (Figure 4c; Figures S2 and S3, and Video S2, Supporting Information). These visualizations provide additional substantiation for the aforementioned conclusions. Evident within the surface region, Cases VI and VII display localized significant deformation of the CBD+E domain (defined as the homogenized domain composed of CBD and electrolyte, see Experimental Section), effectively illustrating the larger deformation encompassing the entire electrode in these two cases. Upon scrutinizing the local stress distribution, a nontrivial trend emerges where Cases VI and VII exhibit less favorable local stress conditions compared to the other cases. This discrepancy can be attributed to two primary factors: 1) The CBD+E domain with reduced mechanical robustness, accommodating particles featuring larger radial gradients, gives rise to pronounced stress conditions at the particle surface within the upper domain. This heightened stress situation may potentially trigger the debonding failure between particles and CBD, induced by significant tensile stress acting tangentially at the particle surface; and 2) The more robust CBD+E domain together with the higher lithiation state of SiO particles results in elevated contact stress within the lower domain. This elevated stress can contribute to particle cracking due to increased compressive stress or instigate particle debonding owing to substantial tensile stress formation during the delithiation process along the radial direction, especially after several cycles.

In summary, opting for a smaller CBD proportion within the upper domain compared to the lower domain holds the potential to enhance electrochemical performance, particularly concerning Li plating behavior. However, this strategic choice also introduces the possibility of mechanical failures (e.g., particle‐CBD debonding), which demands due consideration in the design process.

## 3 Discussion

### 3.1 Coupling Effects of SiO Locations and CBD Inhomogeneities

**Figure 5:**
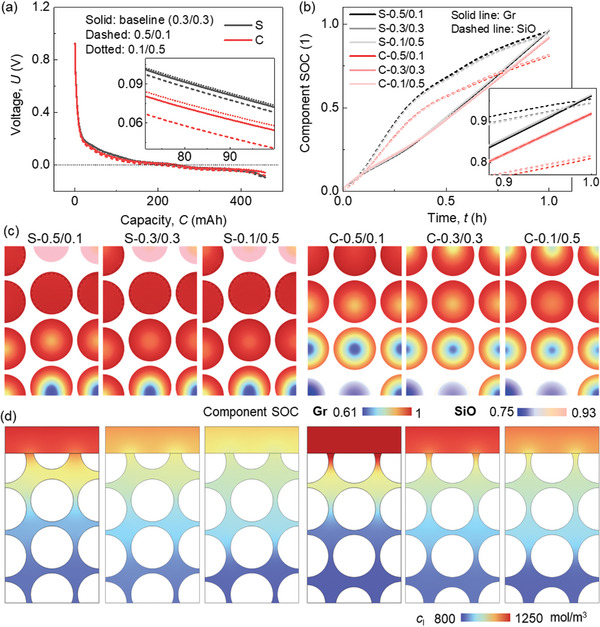
Computation results of the SiO/Gr composite anode (SiO ≈5%) with coupled effects of CBD inhomogeneities (three representatives: 0.5/0.1, 0.3/0.3, and 0.1/0.5) and SiO locations during the lithiation process about a) voltage profile; b) component SOCs of SiO and Gr materials; c) the component SOCs and d) liquid phase concentration distributions at the end of lithiation (3600 s). (S: SiO distributed near the separator; C: SiO distributed near the current collector).

The half‐cell voltage profiles exhibit a consistent trend, as elucidated in the Results Section, for both scenarios involving the distribution of SiO particles, whether they are located near the separator (case‐S) or the current collector (case‐C) (**Figure** **5**a). However, the CBD inhomogeneity effect in case‐C is larger than that in case‐S. For the component SOC profiles (Figure 5b), the changing tendency in case‐S is same as the typical results discussed in Results Section. However, the component SOCs of both SiO and Gr are improved in case‐C when the CBD proportion is smaller in the upper domain than that in the lower domain. This is because compared to the baseline models, the SiO particles are all distributed in the lower domain where the CBD proportion is increased, thus facilitating the lithiation of all the SiO particles. The SOC distribution analysis within case‐S reveals a consistent pattern with the typical results in Results Section within the lower domain: a reduction in the Li^+^ gradient within Gr particles with increasing CBD proportion (Figure 5c; Video S3, Supporting Information). In the upper domain, a pronounced gradient expansion occurs solely in SiO particles as the CBD proportion decreases. Conversely, all Gr particles in this domain exhibit a phenomenon of surface over‐lithiation. This phenomenon can be predominantly attributed to the placement of SiO particles close to the separator, resulting in heightened activity within the upper domain owing to substantial lithiation in the SiO particles. Consequently, this accelerates the complete lithiation of adjacent Gr particles, leading them to be over‐lithiated by the conclusion of the lithiation process. As for the component SOC distribution in case‐C, the tendency is the same as discussed in Results Section both for the upper domain and lower domain. The distribution of liquid phase Li^+^ concentrations follow a consistent trend with the typical results wherein a reduction of CBD proportion in the upper domain and its augmentation in the lower domain lead to smaller polarization within the electrolyte (Figure 5d). Notably, this enhancement is more pronounced in case‐C than in case‐S, despite the fact that the gradient's absolute value in each CBD inhomogeneity of case‐S is smaller when compared to the corresponding one from case‐C.

**Figure 6:**
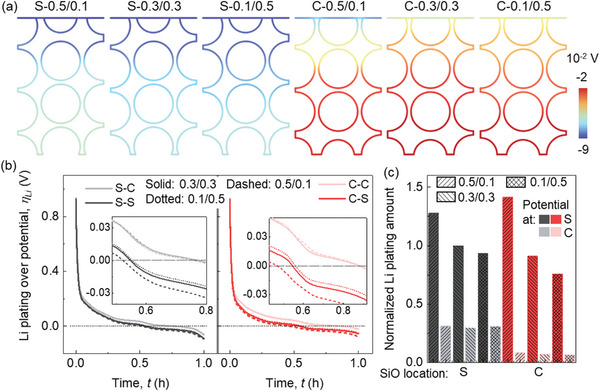
Computation results of the SiO/Gr composite anode (SiO ≈5%) with coupling effects of CBD inhomogeneities (three representatives: 0.5/0.1, 0.3/0.3, and 0.1/0.5) and SiO locations during the lithiation process about a) Li plating overpotential distribution at the end of the lithiation process; b) the evolution of the Li plating overpotentials during the lithiation process at one point near the separator and the other one near the current collector (Legend Explanation: for example “S‐C”, the character preceding the dashed line signifies the placement of SiO, with “S” denoting proximity to the separator (“C” signifying proximity to the current collector). The character following the dashed line indicates the position of the Li plating overpotential, with “C” referring to the surface situated between the current collector and the anode coating layer (“S” indicating the surface located between the separator and the anode coating layer).); c) the Li plating amount (normalized by the Li plating amount near the separator of baseline case‐S (case‐S‐0.3/0.3)).

The overpotentials of Li plating in case‐S are generally more negative than those in case‐C with the same CBD inhomogeneity at the end of lithiation process (**Figure** **6**a; Video S3, Supporting Information). In all scenarios, a consistent pattern emerges, wherein the overpotential near the separator registers a more negative trend. Notably, the trend of the Li plating overpotential closely aligns with that of the half‐cell voltage. In case‐C, the impact of CBD inhomogeneity is particularly pronounced for positions adjacent to the separator, while the overpotential close to the current collector remains relatively less affected by CBD inhomogeneity for both case‐S and case‐C (Figure 6b). In situations where the CBD proportion in the upper domain surpasses that in the lower domain, positioning SiO in proximity to the separator yields a reduction in Li plating within the upper domain, while concurrently encouraging deteriorated Li plating within the lower domain (Figure 6c). Conversely, across other scenarios, the strategic placement of SiO near the separator consistently promotes increased Li plating, observable both near the separator and the current collector. Consequently, regardless of the SiO specific placement, the strategic decision to maintain a smaller CBD proportion within the upper domain compared to the lower domain proves advantageous in mitigating Li plating. This outcome can be attributed to the fact that a reduced CBD proportion within the upper domain can lead to diminished polarization along the thickness direction, thereby facilitating a more uniform distribution of potential. This, in turn, helps mitigate the occurrence of over‐lithiated particles, given that the upper domain inherently possesses greater electrochemical activity, even when the CBD is uniformly distributed.

**Figure 7:**
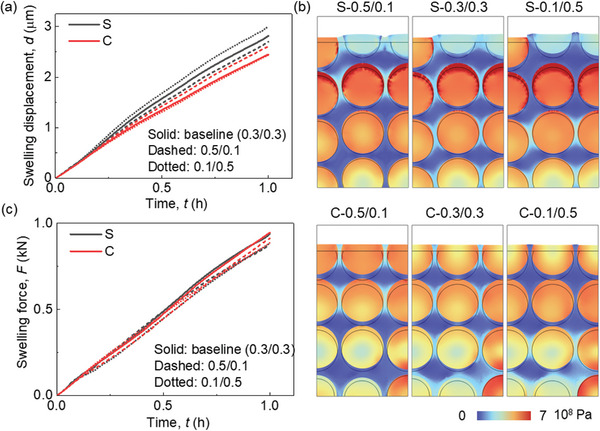
Comparison of mechanical behaviors among models with three representative CBD inhomogeneities (0.5/0.1, 0.3/0.3, and 0.1/0.5) in SiO/Gr anode (SiO w ≈ 5%) considering SiO locations about the a) average thickness change; b) Von Mises stress distribution and the deformation (the frame shows the original shape) at the end of the lithiation process (3600 s); c) average swelling force in the in‐plane direction.

The overall lithiation‐induced deformation in case‐S is larger than that in case‐C for all the CBD inhomogeneities due to the distribution of large expansion SiO particles at the top surface, consistent with our previous study^[^ ^39^ ^]^ (**Figure** **7**a). The case‐S‐0.1/0.5 shows the most pronounced deformation across all three instances of case‐S. This outcome primarily stems from the influence of the softer CBD+E domain where the CBD proportion is less than that in the other two cases (Figure 7b). Notably, there is an obvious restraining effect exerted by the CBD+E domain upon the SiO particles within cases S‐0.5/0.1. Conversely, a contrasting trend unfolds within all instances of case‐C, wherein a reduction in CBD proportion corresponds to a decrease in overall deformation. This difference can be attributed to the prevailing factor controlling the overall anode deformation being the deformation of Gr particles in the upper domain, as opposed to the deformation of CBD+E in case‐S. The diminishment of CBD proportion within the upper domain curtails the lithiation within the Gr particles contained within that specific domain, thereby resulting in an overall reduction in deformation. Given that Gr particles exhibit comparatively modest and uniform swelling in contrast to SiO, the deformation of the CBD+E domain caused by the swelling of the contained Gr particles exerts minor influence on the overall deformation patterns (Figure 7b). The greatest magnitude of the overall swelling force along the in‐plane direction is exhibited in the uniform CBD case (0.3/0.3) for both case‐S and case‐C. Conversely, the other two CBD inhomogeneities (0.5/0.1 and 0.1/0.5) demonstrate comparable force magnitudes (Figure 7c). This trend aligns with the observations outlined in the Results Section, wherein the swelling force initially increases and subsequently decreases as the CBD inhomogeneity transitions from 0.5/0.1 to 0.1/0.5. Thus, it can be concluded that this in‐plane swelling force evolution is mainly influenced by the detailed particle stress change. It is important to note that the overall deformation of the electrode is not a direct indicator of the swelling force change. Although case‐S and case‐C show competitive global swelling force magnitudes, the localized stresses demonstrate obvious difference (Figure 7b; Figures S4 and S5, and Video S4, Supporting Information). The Gr particles of the second layer in the upper domain exhibit localized stress concentration at particle surface due to over‐lithiation for all the case‐S. Higher CBD contents in the upper domain enhance the CBD+E domain's capacity to manage stress and SiO deformation (case‐S‐0.5/0.1). Conversely, lower CBD contents make the CBD+E domain less effective at sustaining SiO deformation, resulting in increased stress concentration on neighboring Gr particle's surface due to SiO particle movement (case‐S‐0.3/0.3 and S‐0.1/0.5). In the lower domain of all case‐S instances, stress patterns remain uniform due to shallower lithiation levels compared to the upper domain. In all case‐C scenarios, both the upper and lower domains exhibit relatively even stress distributions in comparison to case‐S. This is attributed to the positioning of SiO near the current collector, which leads to reduced lithiation in both SiO and Gr particles, resulting in similar stress levels for both materials. However, the combination of the stiffer CBD+E domain with higher CBD contents in the lower domain, coupled with large stress and deformation in SiO particles, culminates in an exceptionally adverse stress condition affecting the neighboring Gr particle at the same thickness layer (as depicted by the Gr particle in the lower right corner of Figure 7c).

Previous studies^[^ ^39^ ^]^ have recommended locating SiO close to the separator in cases of uniformly distributed CBD content. However, this study proposes that increasing the CBD proportion in the lower domain yields enhanced performance for anodes with SiO situated near the current collector, both from electrochemical and mechanical standpoints. While the analysis in the Results Section and for case‐C advocates for improved performance with a higher CBD proportion in the lower domain, this approach is not suitable for case‐S. To ensure mechanical robustness of the anode, a larger CBD proportion in the upper domain is suggested when SiO is positioned there. In conclusion, when deliberately determining SiO placement in the anode design process, it is recommended to incorporate a higher CBD proportion in the same region for optimal outcomes.

### 3.2 CBD Inhomogeneity Effects on Rate Performance

**Figure 8:**
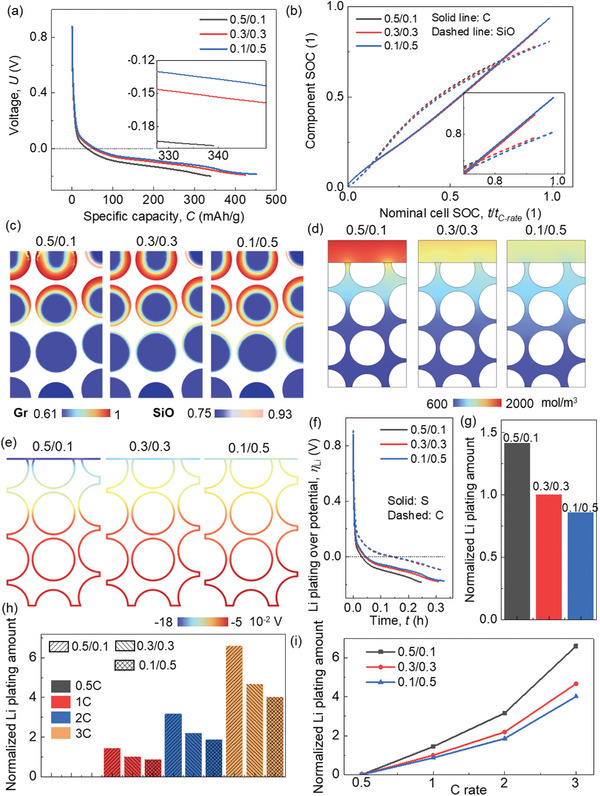
Computation results of the SiO/Gr composite anode (SiO ≈5%) with various CBD inhomogeneities (three representatives: 0.5/0.1, 0.3/0.3, and 0.1/0.5) under high C rate (3C) about a) voltage profile; b) component SOCs of SiO and Gr materials; c) the component SOCs, d) liquid phase concentration, and e) Li plating overpotential distributions at the end of lithiation (≈886 s); f) the evolution of the Li plating overpotentials during the lithiation process at one point near the separator and the other one near the current collector (S: near separator, C: near current collector); g) the Li plating amount (normalized by the baseline case (case‐0.3/0.3)). Comparison among various C‐rates about the Li plating amount in the format of h) histogram and i) line chart (all normalized by the baseline case (case‐0.3/0.3) at 1C rate).

Here, results in 3C rate are selected to show the basic electrochemical and mechanical behavior. Among these scenarios, the instance with a lower CBD proportion in the upper domain (0.1/0.5) exhibits a notably competitive performance in terms of voltage behavior, manifesting a relatively higher voltage level and attainable capacity (**Figure** **8**a). This effect is particularly pronounced when subjected to high charging rates (Figure S6, Supporting Information). Specifically, in the case of 0.1/0.5, there is a distinct augmentation in the real SOC for both the cell and its constituents under high C rates (Figure 8b; Figure S7). Delving into more intricate details, the distribution of Li^+^ concentration reveals a surprisingly pronounced state of over‐lithiation within the 0.5/0.1 configuration, occurring around the nominal cell SOC of ≈0.8 during high C rates. This contrast becomes particularly evident when comparing against lower C rates (Figures 8c 2c; Figure S8a,c, Supporting Information), which in turn hampers the progression of further lithiation. Notably, such a phenomenon is effectively mitigated within the 0.1/0.5 case through the reduction of electrochemical activity within the upper domain. Consequently, this configuration showcases diminished polarization within the electrolyte, as evidenced by the data presented in Figure 8d and Figure S8b,d (Supporting Information).

The gradient of Li plating overpotential exhibits a more pronounced variation across all scenarios during high C rates as opposed to lower C rates (Figures 8e, 3a, and Figure S9, Supporting Information). Analyzing the evolution profiles of Li plating overpotential, it becomes evident that CBD inhomogeneity exerts minimal influence on Li plating behavior in proximity to the current collector (Figure 8f). However, as the charging rate increases, the impact of CBD inhomogeneity on Li plating behavior adjacent to the separator becomes more conspicuous (Figure 8f; Figure S10a,b, Supporting Information). In the context of 3C rate lithiation, the lowest Li plating amount is observed in case‐0.1/0.5, a trend consistent with results from lower C rates. Furthermore, a comparative analysis of the total Li plating quantities across various C rates reveals that case‐0.1/0.5 outperforms other configurations, particularly evident under high C rates (Figure 8h,i). Consequently, the strategy of reducing the CBD proportion within the upper domain to a level below that in the lower domain exhibits the potential to enhance rate performance (a proposition also supported by Figures S11 and S12, Supporting Information).

**Figure 9:**
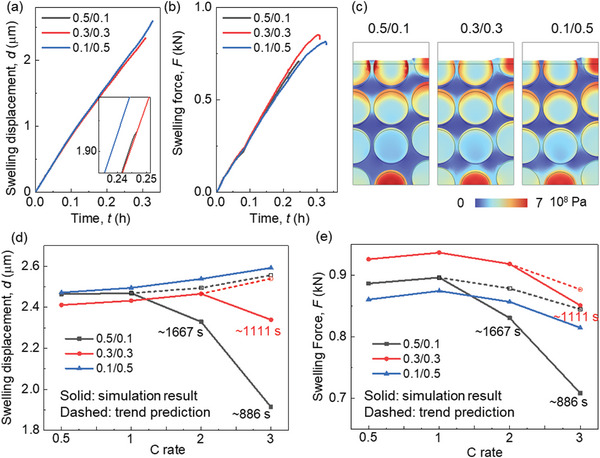
Comparison of mechanical behaviors among models with three representative CBD inhomogeneities (0.5/0.1, 0.3/0.3, and 0.1/0.5) in SiO/Gr anode (SiO w ≈ 5%) considering various C rates about the a) average thickness change under 3C rate; b) average swelling force in the in‐plane direction under 3C rate; c) Von Mises stress distribution and the deformation (the frame shows the original shape) near the end of the lithiation process (≈886 s) under 3C rate; comparison of d) the maximum anode deformation and e) in‐plane swelling force among the three CBD inhomogeneities under four C rates.

Among the three CBD inhomogeneity scenarios, the most notable overall anode deformation during 3C rate lithiation occurs in case‐0.1/0.5, as depicted in **Figure** **9**a. This outcome is attributed to the more favorable lithiation conditions within the upper domain of this configuration in comparison to the other two (Figure 8c), resulting in a more thorough lithiation process and consequently, a greater deformation. However, it's important to note that the in‐plane average swelling force is relatively smaller for case‐0.1/0.5 at the same lithiation duration (Figure 9b). Detailed stress distribution further underscores the superior stress conditions at approximately the same lithiation stage (≈SOC 80%) in case‐0.1/0.5 (Figure 9c). Additionally, the localized stress concentration at the surface of the graphite (Gr) particles within the upper domain transpires at later stages in case‐0.1/0.5 and case‐0.3/0.3, when compared to case‐0.5/0.1. This divergence is especially pronounced under high C rates (Figures S13–S15, Supporting Information). Furthermore, summarized in Figure 9d,e are the maximum swelling deformation and swelling force across different C rates, respectively. Notably, since the lithiation processes within case‐0.5/0.1 and case‐0.3/0.3 terminate before the theoretically complete lithiation time for high C rates (1800 s for 2C and 1200 s for 3C), trend predictions are derived by analyzing deformation and stress evolution (as shown in Figure S16, Supporting Information). These analyses offer deeper insights into the rate performance across all CBD inhomogeneity cases. Overall, the outcomes demonstrate that the maximum swelling displacement ideally increases with increasing C rates for all scenarios. In terms of in‐plane swelling force, an initial rise followed by a subsequent decline is evident, with the maximum value observed at 1C rate. The data indicates that case‐0.5/0.1 strikes a balance by yielding relatively moderate deformation and in‐plane swelling force. However, considering the electrochemical performance, case‐0.1/0.5 is the more recommended choice due to its lower in‐plane swelling force. Additionally, the variation in deformation among the three cases under different C rates remains relatively modest. Furthermore, it's worth highlighting that the detailed stress condition within case‐0.1/0.5 is notably superior to that of case‐0.5/0.1 (Figures S13–S15, Supporting Information).

## 4 Conclusion

During the drying process of lithium‐ion battery electrode manufacturing, an inhomogeneity of the distribution of carbon‐binder domain (CBD) could be formed, which may influence the battery performance from both electrochemical and mechanical perspectives. A comprehensive electrochemical‐mechanical coupled model incorporating particle geometries has been formulated. This model aims to investigate the repercussions of CBD inhomogeneity on battery performance, utilizing a SiO/Gr composite anode material as its basis. A limitation should be noted that the 2D simplification of the current model may result in the neglect of the third dimension concerning material properties and physical behaviors, such as diffusion and expansion. Various anodes exhibiting distinct CBD inhomogeneities were subjected to sophisticated electrochemical behavior analysis. This encompassed a comprehensive assessment of voltage profiles, evolution of component state of charge (SOC), localized distributions, electrolyte polarization, and Li plating. The swelling force and deformation evolutions and detailed distributions were discussed for the mechanical aspect as well. The coupling effects of the SiO locations and the CBD inhomogeneities were then analyzed, followed which the rate performance considering the CBD inhomogeneities was examined.

In summary, the following conclusions can be drawn:

- Decreasing the CBD proportion in the upper domain to a level smaller than that in the lower domain yields a more homogenous Li^+^ concentration distribution. Consequently, electrolyte polarization is diminished, and the occurrence of Li plating is minimized. Nevertheless, this attenuation of the CBD in the upper domain introduces a potential susceptibility to mechanical breakdown at the interfaces between particles and CBD within the upper domain.
- When contemplating a manual design of SiO distribution, a strategic placement of the higher CBD proportion alongside SiO particles is advisable. This approach underscores that positioning SiO near the current collector, coupled with a higher CBD proportion in the lower domain, cultivates a balanced environment, curbing Li plating while scarifying some capacity. Conversely, an increased CBD proportion in the upper domain along with SiO situated closer to the separator yields significant capacity, albeit accompanied by heightened polarization, pronounced Li plating, and intensified stress concentrations.
- Diminishing the CBD proportion within the upper domain is further corroborated to yield favorable outcomes for rate performance, spanning both electrochemical and mechanical considerations.

This study introduces a comprehensive modeling framework aimed at elucidating the implications of CBD inhomogeneity within SiO/Gr composite anodes. By establishing a foundational knowledge of the interrelated behaviors, the findings offer valuable insights into the design and fabrication of SiO/Gr composite materials for advanced, high‐energy‐density anodes in the next generation. Moreover, this model has the potential for future extensions to encompass more realistic microstructure, incorporating non‐spherical particles, 3D data, and more rigorous validation processes, aiming to broaden its application, thus providing more insights into the design of SiO/Gr anodes.

## 5 Experimental Section

### Multiphysics Modeling

In this model, two physics are considered: electrochemical field and mechanical field. They were coupled by transferring key parameters between them with some reasonable assumptions. The details are described in the following parts.

### Electrochemical Field

The electrochemical behaviors of the electrode mainly include the intercalation in the active particles and the reaction at the particle surface according to the theory of Newman's battery model (**Table** **1**).

**Table 1:** Equations of the Newman's battery model.

Electrode level		
Current density in liquid phase	$i_{l}=-\kappa_{l}^{eff}\left[\nabla \phi_{l}-\frac{2RT}{F}\left(1+\frac{d\ln f_{\pm }}{d\ln c_{l}}\right)\left(1-t_{+}\right)\nabla \ln c_{l}\right]$	(1)
Current density in solid phase	$i_{s}=-\kappa_{s}^{eff}\nabla \phi_{s}$	(2)
Li^+^ flux density in liquid phase	$J_{l}=-D_{l}^{eff}\nabla c_{l}+\frac{t_{+}}{F}i_{l}$	(3)
Charge conservation	∇ · **i**_*l*_ = *a_s_i*, ∇ · **i**_**s**_ = −*a_s_i*	(4)
Mass conservation	$\varepsilon_{l}\frac{\partial c_{l}}{\partial t}=-\nabla \cdot J_{l}+\frac{a_{s}i}{F}$	(5)

Particle level		
Li‐ion intercalation/deintercalation	$\frac{\partial c}{\partial t}+\nabla \cdot J=0$, $J=\frac{D_{eff}}{RT}\left(FK\nabla c+\Omega c_{\max}\nabla \sigma_{h}\right)$	(6)

Interface		
Local current density	$i=i_{0}\left\{\exp\left[\frac{(1-\beta )F\eta }{RT}\right]-\exp\left(-\frac{\beta F\eta }{RT}\right)\right\}$	(7)

The initial conditions and boundary conditions are listed here (the subscript SiO and Gr represent the parameters in the corresponding materials, respectively in the following descriptions):

(8) $$c_{\mathrm{SiO}}=c_{\mathrm{SiO},0},c_{\mathrm{Gr}}=c_{\mathrm{Gr},0}\;\mathrm{at}\;t=0$$

(9) $$J\cdot \upsilon =-\frac{i}{F}\;\mathrm{at}\;\mathrm{boundary}\;\Gamma$$

where *i* is the local current density. It is calculated from the interfacial reaction at the active particle and electrolyte interface according to the B–V (Butler–Volmer) function (Equation 7) with the exchange current density as

(10) $$i_{0}=Fk_{c}^{\alpha_{a}}k_{a}^{\alpha_{c}}{\left(c_{\max}-c_{\mathrm{surf}}\right)}^{\alpha_{a}}{\left(c_{\mathrm{surf}}\right)}^{\alpha_{c}}{\left(c_{l}/c_{l,\mathrm{ref}}\right)}^{\alpha_{a}}$$

The boundary here is the surface of each particle in touch with the electrolyte. Thus, Equation (7) bridges the electrochemical reactions (Equations (1–5) at electrode level and the Li‐ion intercalation (Equation 6) at the particle level.

The Li plating is also theoretically analyzed. The overpotential of the Li plating is considered by η_*Li*_ = ϕ_*s*_ − ϕ_*l*_. Then, the Li plating energy is used to quantitatively represent the total amount of the Li plating:

(11) $$E_{\mathrm{plating}}=\int_{t_{1}}^{t_{2}}\left|\eta_{Li}\right|Idt$$

where *t*_1_ and *t*_2_ indicate the period of Li plating (Li plating is initiated when η_*Li*_ = ϕ_*s*_ − ϕ_*l*_<0), and *I* is the anode current.

### Mechanical Field

The mechanical behaviors of active particles (Gr and SiO) and the CBD were considered. Both elastic and plastic behaviors were included in the proposed model. For the equilibrium equation of the elastic stage, it is written by

(12) ∇·T+B=0

where ***T* = C:E** is the nominal stress (**C** is the stiffness matrix and **E** is elastic strain matrix), and **B** is the body force which equals zero here. Von Mises law is used as the yield law for the plastic stage. The perfect plasticity model is applied for the hardening stage with an initial yield strength related to the Li‐ion concentration for SiO material, while a linear plasticity model is introduced for the CBD.

According to the multiplicative decomposition law, the total deformation gradient can be written as

(13) $$F=F_{e}\cdot F_{p}\cdot F_{l}$$

where **F** represents the deformation gradient and the subscripts “**e**,” “**p**,” and “**l**” represent elastic, plastic, and lithiation‐induced, respectively. The elastic and plastic deformation are calculated by the elastic and plastic models mentioned above, respectively, while the lithiation‐induced volumetric deformation, **F_l_**, is related to the Li^+^ concentration in the solid phase, *c*. This lithiation‐induced swelling is assumed to be isotropic, thus the relationship is expressed as $F_{l}=\frac{\Omega }{3}\Delta c$.

### Coupling Strategy

**Figure 10:**
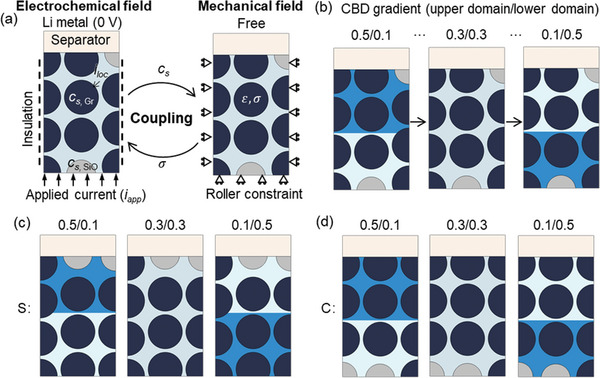
a) The coupling strategy and the boundary conditions of the RVE model; b) The setups of the inhomogeneity of CBD; coupling effects of the CBD inhomogeneity and SiO positions in the scenarios of SiO distributed near c) separator and d) current collector.

The diffusion model and mechanical model were coupled by transferring the hydrostatic stress *σ*_h_ and the Li^+^ concentration *c* between the two models as Equations (6) and (13) indicate (**Figure** **10**a). The hydrostatic stress, *σ*_h_ is defined as the mean value of three principal stresses, $\frac{\left(\sigma_{1}+\sigma_{2}+\sigma_{3}\right)}{3}$, which is calculated by the mechanical model. This hydrostatic stress influences the diffusion process within the particles according to the second term of Equation (6), named as the stress‐driven diffusion. This is how mechanical fields affect the electrochemical field. Contrarily, the diffusion of Li^+^ within the particles triggers a notable volume change, subsequently resulting in the stress evolution within and among particles and the CBD.

In the present study, certain assumptions are made during the coupling of the models: First, the stress‐induced overpotential change was disregarded due to its magnitude being approximately two orders of magnitude smaller than the range of overpotential observed in the target scenario, as per the pre‐calculation. Second, only the stresses were transferred from the mechanical field to the electrochemical field, while the strain is intentionally not transferred to prevent any pressure effects on the electrolyte flows. Finally, the impact of volumetric change on the diffusion process was taken into account by introducing a coefficient in Equation (6) as *V*_1_/*V*_0_, where *V*_0_ represents the initial volume and *V*_1_ represents the expanded volume, respectively.

### Finite Element Computation and Parametric Setups

A 2D representative volume element (RVE) considering the detailed particle description was extracted from the anode layer along the thickness direction to serve as the target for the subsequent numerical studies (Figure 1b). In practical anodes, the active particles exist in two representative formats: they were either wrapped by the CBD or in direct contact with surrounding particles. In both cases, a valid pathway for electron transport was established, ensuring effective electron conduction within the anode. In the present study, direct contact between particles was not considered. Instead, all the particles were effectively wrapped by the CBD layer (The total CBD domain comprises ≈10% of the total volume in all the models considered in this study). The RVE model contains one column of complete particles and two columns of half‐particles with a convergence study performed to confirm the RVE size (Figures S17–S19, Supporting Information). A half‐cell configuration was considered, with Li metal, separator, and anode layer in the sequence along the thickness direction. The anode layer was composed of active particles (SiO and Gr), CBD, and electrolytes. The CBD and electrolyte were treated as a single domain (named as CBD+E), accounting for ≈36% of the total volume in all the models considered in this study. This combined domain serves the dual function of facilitating both electron and ion transportation.^[^ ^51^, ^52^ ^]^ The distribution of the SiO particles was manually determined in the baseline model. About 1500 Triangular elements were used in the model. Despite the changes in volume depicted in the model, there is no need for remeshing.

For the baseline model, the CBD proportion in the CBD+E domain was uniformly distributed and set as 0.3. To mimic the inhomogeneity of the CBD distribution, the CBD+E domain was divided into two sub‐domains with identical volume: upper domain (near the separator) and lower domain (near the current collector). This method reduces the computational complexity and is practically achievable (e.g., two‐layer coating methodology). Consequently, the baseline case was defined as 0.3/0.3, signifying that the CBD fractions in both the upper and lower domains were set at 0.3. Then, the inhomogeneity could be achieved by setting different CBD fractions for the two sub‐domains. The average CBD fraction in the whole CBD+E was the same among all the cases. Seven different combinations of inhomogeneity (including the baseline) were considered in this study (Figure 10b; **Table** **2**). For a reference, a pure Gr anode was considered with five different combinations of inhomogeneity (Table 2). The two SiO particles in the SiO/Gr anode were replaced by the Gr particles to generate the pure Gr anode. All the other conditions stay the same.

**Table 2:** Anode formation considering various CBD inhomogeneities.

	Active materials		CBD		Reference code (upper domain/lower domain	Porosity
	Gr	SiO	Upper domain	Lower domain		
Case I	0.59	0.05	0.09	0.018	0.5/0.1	0.252
Case II	0.59	0.05	0.081	0.027	0.45/0.15	0.252
Case III	0.59	0.05	0.072	0.036	0.4/0.2	0.252
Case IV (baseline)	0.59	0.05	0.054	0.054	0.3/0.3	0.252
Case V	0.59	0.05	0.036	0.072	0.2/0.4	0.252
Case VI	0.59	0.05	0.027	0.081	0.15/0.45	0.252
Case VII	0.59	0.05	0.018	0.09	0.1/0.5	0.252
Gr‐Case I	0.64	0	0.09	0.018	0.5/0.1	0.252
Gr‐Case II	0.64	0	0.072	0.036	0.4/0.2	0.252
Gr‐Case III	0.64	0	0.054	0.054	0.3/0.3	0.252
Gr‐Case IV	0.64	0	0.036	0.072	0.2/0.4	0.252
Gr‐Case V	0.64	0	0.018	0.009	0.1/0.5	0.252

For electrochemical boundary conditions, the bottom surface was applied with 1C rate current density *i*_app_, for all the simulation cases, and the top surface was set as the “electrode surface” with properties of Li metal to form the half‐cell configuration. The left and right sides were set as insulation. For the mechanical boundaries, a roller constraint was defined for the bottom, right, and left sides while the top surface was set as free. (Figure 10a) The models were computed in COMSOL platform with an average runtime of 1.5 h with ≈1500 elements in all cases.

### Parametric Setups

In this study, the weight percentage of the SiO particle was constant. The CBD inhomogeneity was one changing parameter. By adjusting the solid content (CBD) proportion of the CBD+E domain, the CBD inhomogeneity could be achieved. In this study, seven different inhomogeneities are considered as mentioned above (Figure 10b; Table 2). In Case I, the upper domain exhibits the highest CBD content, while the lower domain has the lowest CBD content. As we move from Case I to Case IV, the CBD fraction in the upper domain gradually decreases, and simultaneously, it increases in the lower domain until they reach the baseline scenario, where both domains share the same CBD fraction value. Subsequently, from Case IV to Case VII, this trend persists, leading to Case VII having the smallest CBD content in the upper domain but the largest CBD content in the lower domain.

Based on our previous studies,^[^ ^39^, ^53^ ^]^ it has been observed that the SiO location significantly influences cell performance. Consequently, in the current work, the combined effects of CBD inhomogeneity and SiO locations was investigated. Two representative scenarios had devised: SiO distribution near the separator (S) (Figure 10c) and SiO distribution near the current collector (C) (Figure 10d). For each SiO location scenario, three CBD inhomogeneities were considered: large CBD content in the upper domain (0.5/0.1), uniform CBD distribution (0.3/0.3), and small CBD content in the upper domain (0.1/0.5).

All the above‐mentioned parametric cases are applied with 1C rate. Here, three more C‐rates (0.5C, 2C, and 3C) were considered to study the rate performance when considering CBD inhomogeneity. For each C rate, three CBD inhomogeneities same as the settings in the SiO location effects were considered. All the models in this study share the same material properties for SiO, Gr, electrolyte, and binders (a binder material containing carbon black and styrene‐butadiene rubber is considered in this study) (**Table** **3**).

**Table 3:** Input parameters and values in the established multiphysics model.

Parameter	Symbol	Value
RVE width	*W*_RVE_	35.2 µm(estimated)
Anode thickness (single coating)	*L*_an_	56 µm(measured)
Separator thickness	*L*_sep_	10 µm(measured)
Graphite particle radius	*R_Gr_*	8 µm(measured)
Electrical conductivity of Gr^[^ ^54^ ^]^	$\kappa_{s}^{\mathrm{cathode}}$	100 S m^−1^
Electrical conductivity of SiO^[^ ^55^ ^]^	$\kappa_{s}^{\mathrm{anode}}$	1 S m^−1^
Electrical conductivity of CBD	*k*_CBD_	1 S m^−1^ (estimated)
Diffusion coefficient in electrolyte^[^ ^56^ ^]^	*D*_e_	*D_e_* (*c_l_*)
Transference number^[^ ^56^ ^]^	*t*_+_	*t_+_* (*c_l_*)
Transfer coefficient^[^ ^56^ ^]^	α_a_ α_c_	0.5
Partial molar volume of SiO^[^ ^57^ ^]^	Ω_SiO_	4.625 × 10^−6^ m^3^ mol^−1^
Partial molar volume of Gr^[^ ^58^ ^]^	Ω_Gr_	3.17 × 10^−6^ m^3^ mol^−1^
Modulus of SiO^[^ ^59^ ^]^	*E*_SiO_	*E_SiO_* (*c_SiO_*)
Modulus of Gr^[^ ^58^ ^]^	*E*_Gr_	3.85 + 16.446*x* GPa (calculated)
Modulus of the CBD+E domain	*E*_CBD_	2 GPa (estimated according to ref ^[^ ^60^ ^]^)
Plastic model of the CBD+E domain	*/*	Note S1 (Supporting Information)
Maximum Li concentration in SiO^[^ ^55^ ^]^	$c_{SiO,max}$	128 000 mol m^−3^
Maximum Li concentration in Gr^[^ ^61^ ^]^	*c*_Gr,max_	31 507 mol m^−3^
Diffusion coefficient in SiO^[^ ^62^ ^]^	*D*_0,Si_	1.5 × 10^−14^ m^2^ s^−1^
Diffusion coefficient in Gr^[^ ^63^ ^]^	*D*_0,C_	1.4 × 10^−13^ m^2^ s^−1^
Yield stress of SiO^[^ ^64^ ^]^	*σ*_y_ *_, SiO_*	$\sigma_{y,SiO}^{0}-0.4\left(c/c_{\max}\right)\mathrm{GPa}$(modified)
Initial yield stress of SiO^[^ ^64^ ^]^	$\sigma_{y,\mathrm{SiO}}^{0}$	1 GPa (modified)

## Conflict of Interest

The authors declare no conflict of interest.

## Author Contributions

X.G. performed methodology (lead), data curation (lead), formal analysis (lead), and wrote the original draft (lead). J.X. performed conceptualization (lead), supervision (lead), wrote the original draft and reviewed and edited the final manuscript (lead).

## Supporting information

Supporting Information

Supplemental Video 1

Supplemental Video 2

Supplemental Video 3

Supplemental Video 4

## Data Availability

The data that support the findings of this study are available in the supplementary material of this article.
