# Supplementary material for: Carbon Binder Domain Inhomogeneity in Silicon‐Monoxide/Graphite Composite Anode by 2D Multiphysics Modeling
Source: Adv Sci (Weinh). 2024 May 22;11(29):2400729. doi: 10.1002/advs.202400729 (PMC11304268; doi:10.1002/advs.202400729)
Supplement: Supplementary file 1 — Supporting Information [file ADVS-11-2400729-s004.pdf]

ADVANCED SCIENCE
Open Access

Supporting Information

for *Adv. Sci.*, DOI 10.1002/advs.202400729

Carbon Binder Domain Inhomogeneity in Silicon-Monoxide/Graphite Composite Anode by 2D Multiphysics Modeling

*Xiang Gao and Jun Xu**

Supplementary Information

Carbon Binder Domain Inhomogeneity in Silicon-Monoxide/Graphite Composite Anode by 2D Multiphysics Modeling

Xiang Gao, Jun Xu[1*]

Xiang Gao, Jun Xu

Department of Mechanical Engineering, University of Delaware, Newark, DE 19716 USA; Energy Mechanics and Sustainability Laboratory (EMSLab), University of Delaware, Newark, DE 19716 USA

Email: junxu@udel.edu

*Correspondence should be sent to Prof. Jun Xu. Email: junxu@udel.edu

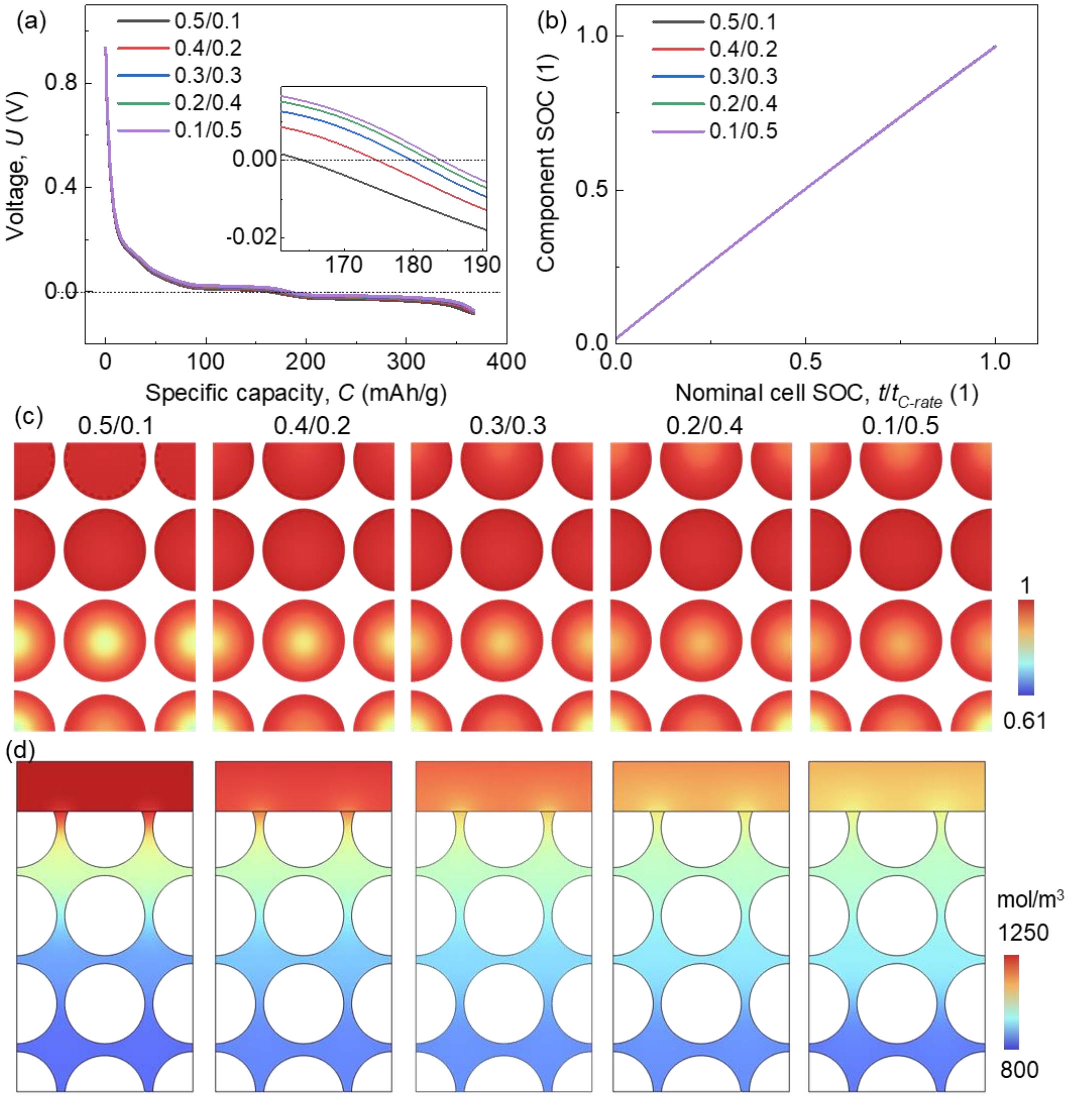

Figure S1. Computation results of the pure Gr anode with various CBD inhomogeneities during the lithiation process about (a) voltage profile; (b) SOC of active Gr particles; (c) the SOC and (d) liquid phase concentration distributions at the end of lithiation.

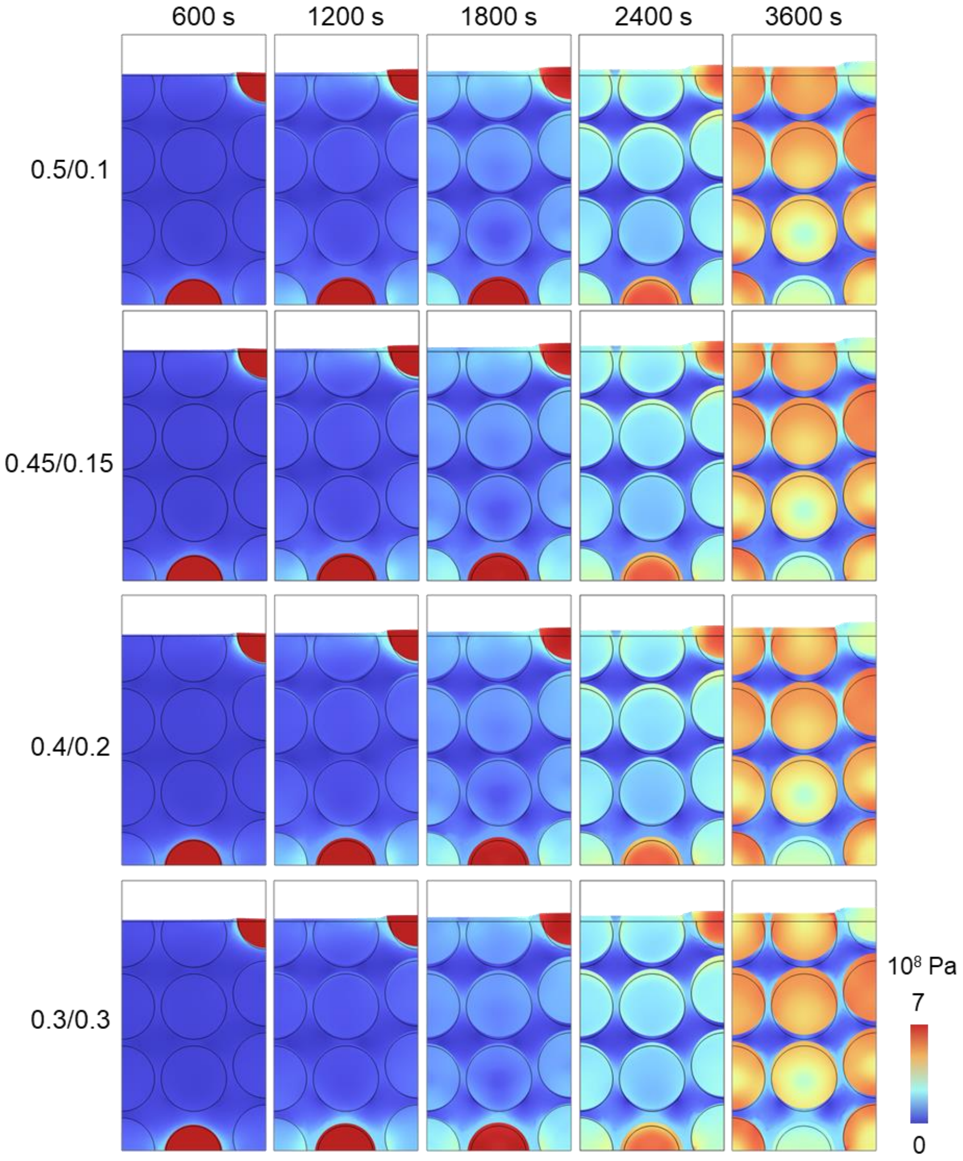

Figure S2. Stress evolution during the lithiation at five selected states in anodes with various CBD inhomogeneities (Case I to Case IV).

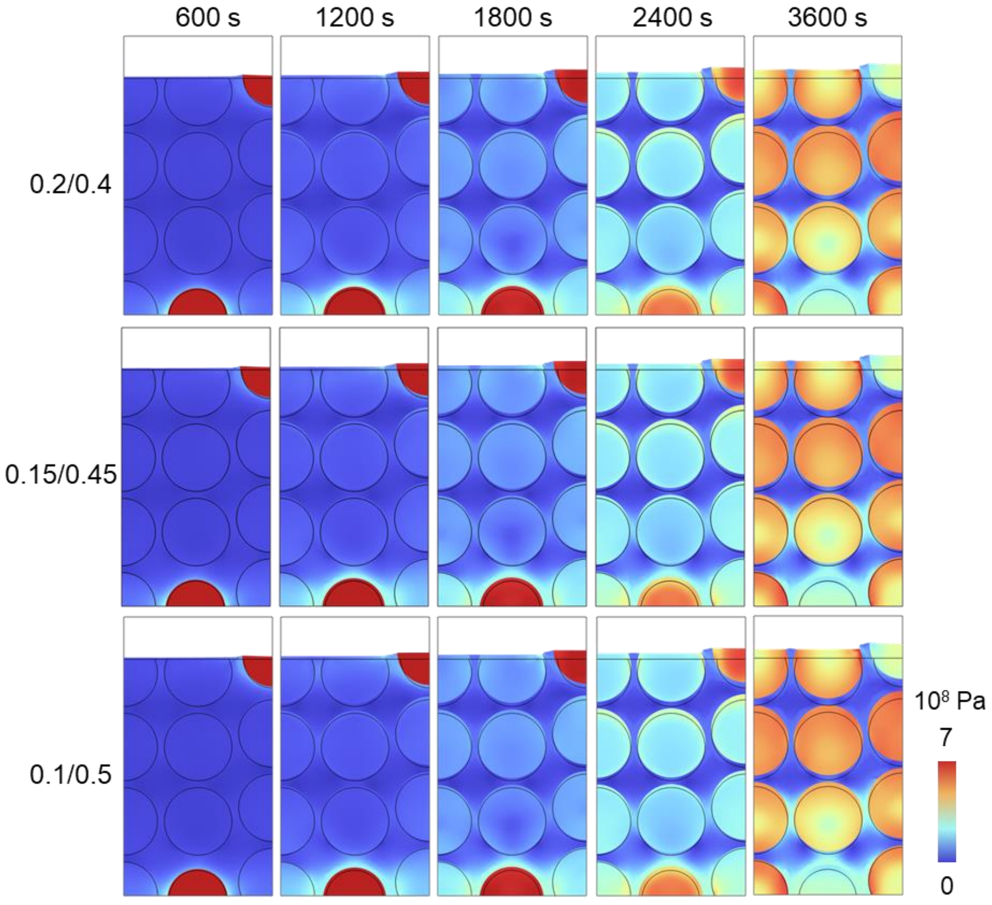

Figure S3. Stress evolution during the lithiation at five selected states in anodes with various CBD inhomogeneities (Case V to Case VII).

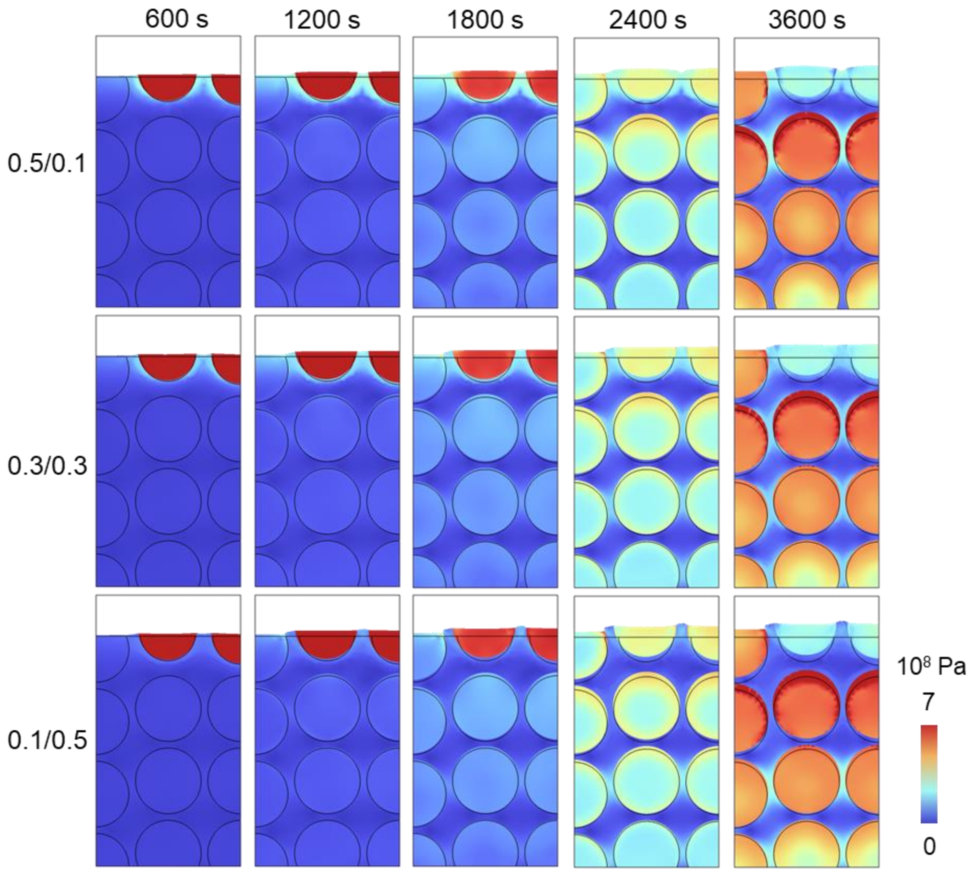

Figure S4 Stress evolution during the lithiation at five selected states in anodes with three representative CBD inhomogeneities considering locating SiO near the separator (case-S).

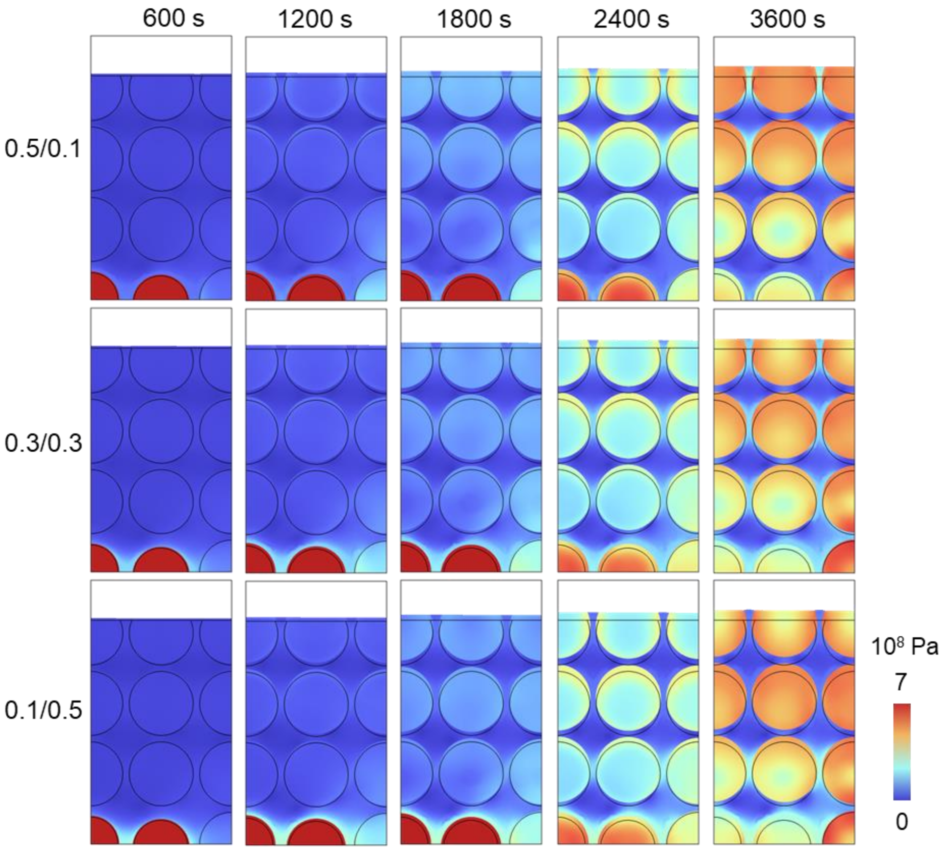

Figure S5. Stress evolution during the lithiation at five selected states in anodes with three representative CBD inhomogeneities considering locating SiO near the current collector (case-C).

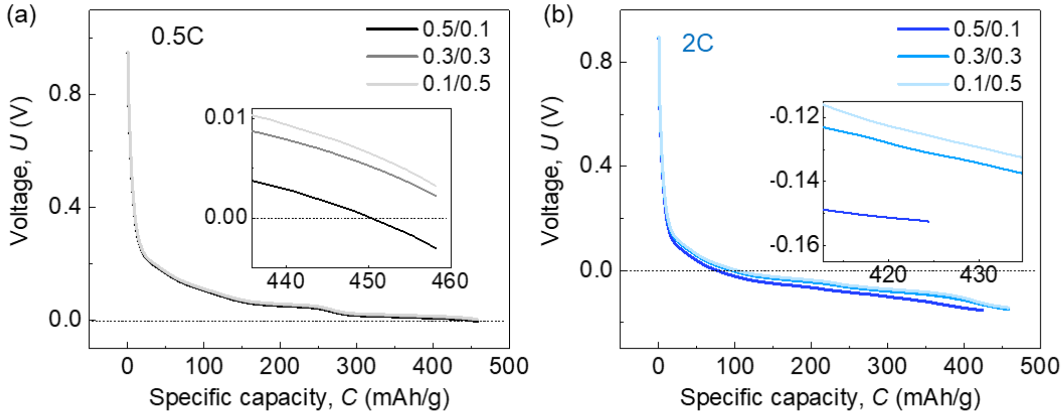

Figure S6. Half-cell voltage profiles of the anodes with three representative CBD inhomogeneities under (a) 0.5C rate and (b) 2C rate conditions.

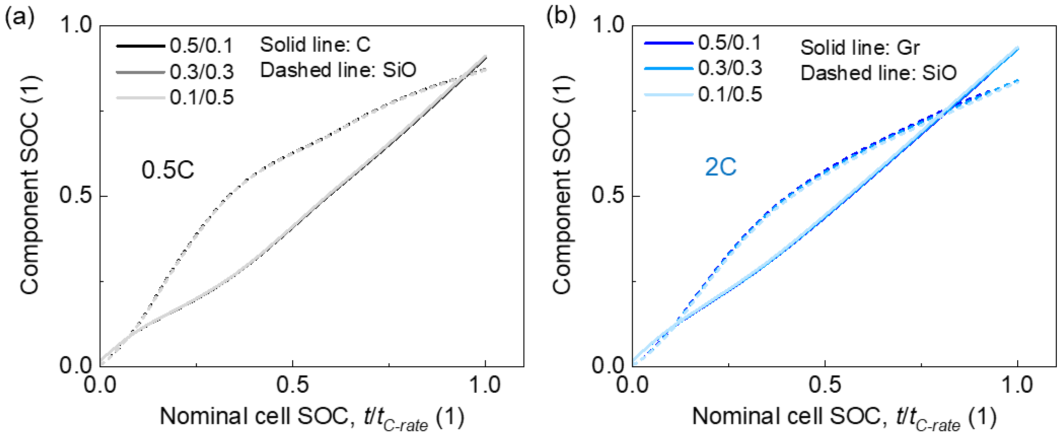

Figure S7. Component SOC profiles of the anodes with three representative CBD inhomogeneities under (a) 0.5C rate and (b) 2C rate conditions.

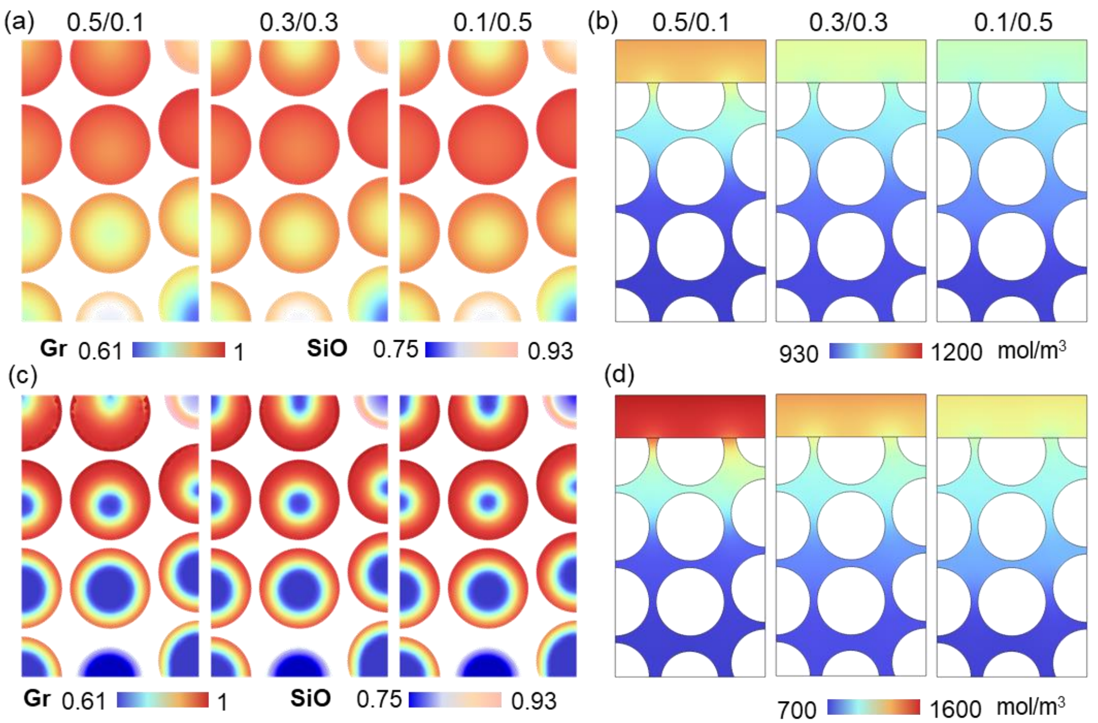

Figure S8. (a) Component SOC and (b) liquid phase Li^+ concentration distributions under 0.5C rate condition, and (c) Component SOC and (d) liquid phase Li^+ concentration distributions under 2C rate condition of the anodes with three representative CBD inhomogeneities at the end of lithiation process.

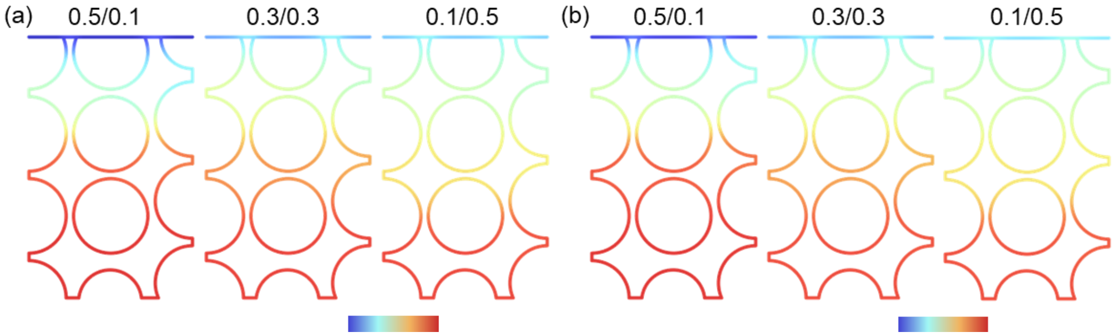

Figure S9. Li plating overpotential distributions of the anodes with three representative CBD inhomogeneities under (a) 0.5C rate and (b) 2C rate conditions.

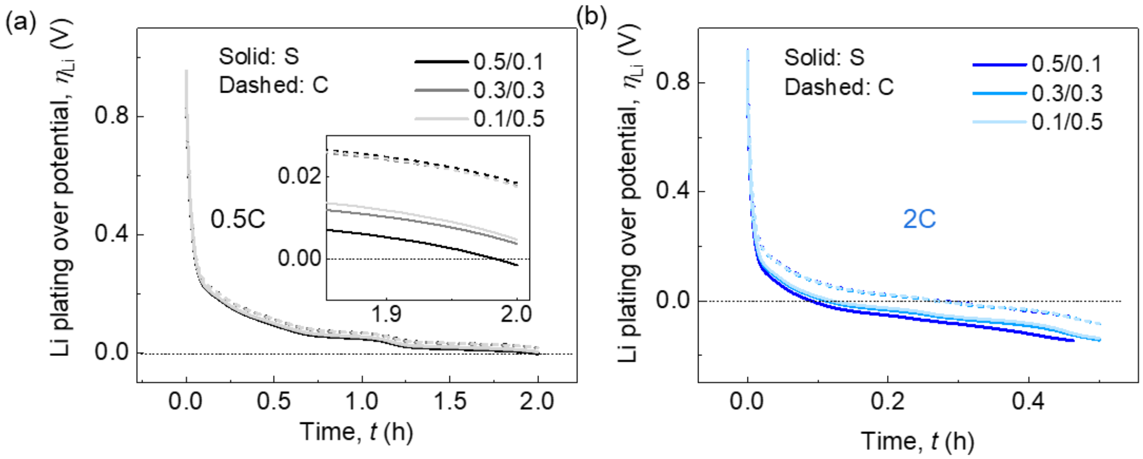

Figure S10. Li plating overpotential evaluation of the anodes with three representative CBD inhomogeneities under (a) 0.5C rate and (b) 2C rate conditions.

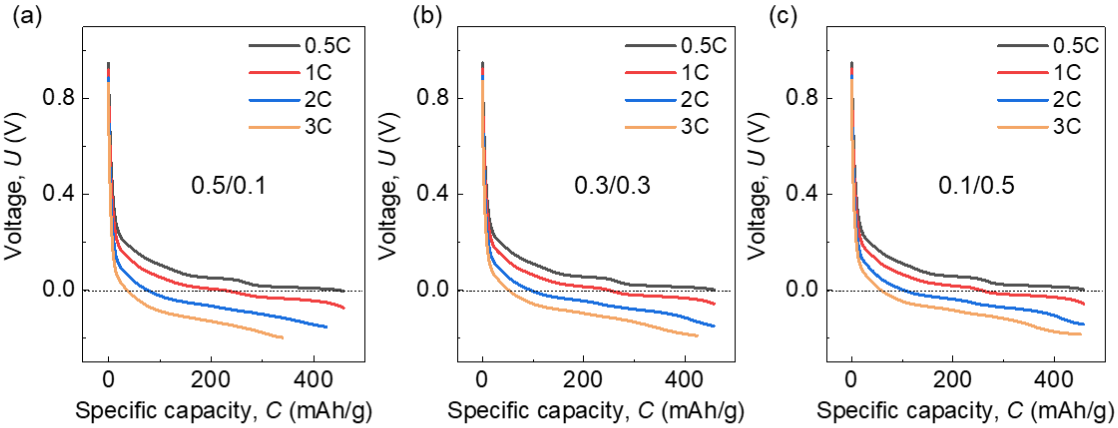

Figure S11. Half-cell voltage profiles under various C rates for the case with CBD inhomogeneity of (a) 0.5/0.1, (b) 0.3/0.3, and (c) 0.1/0.5.

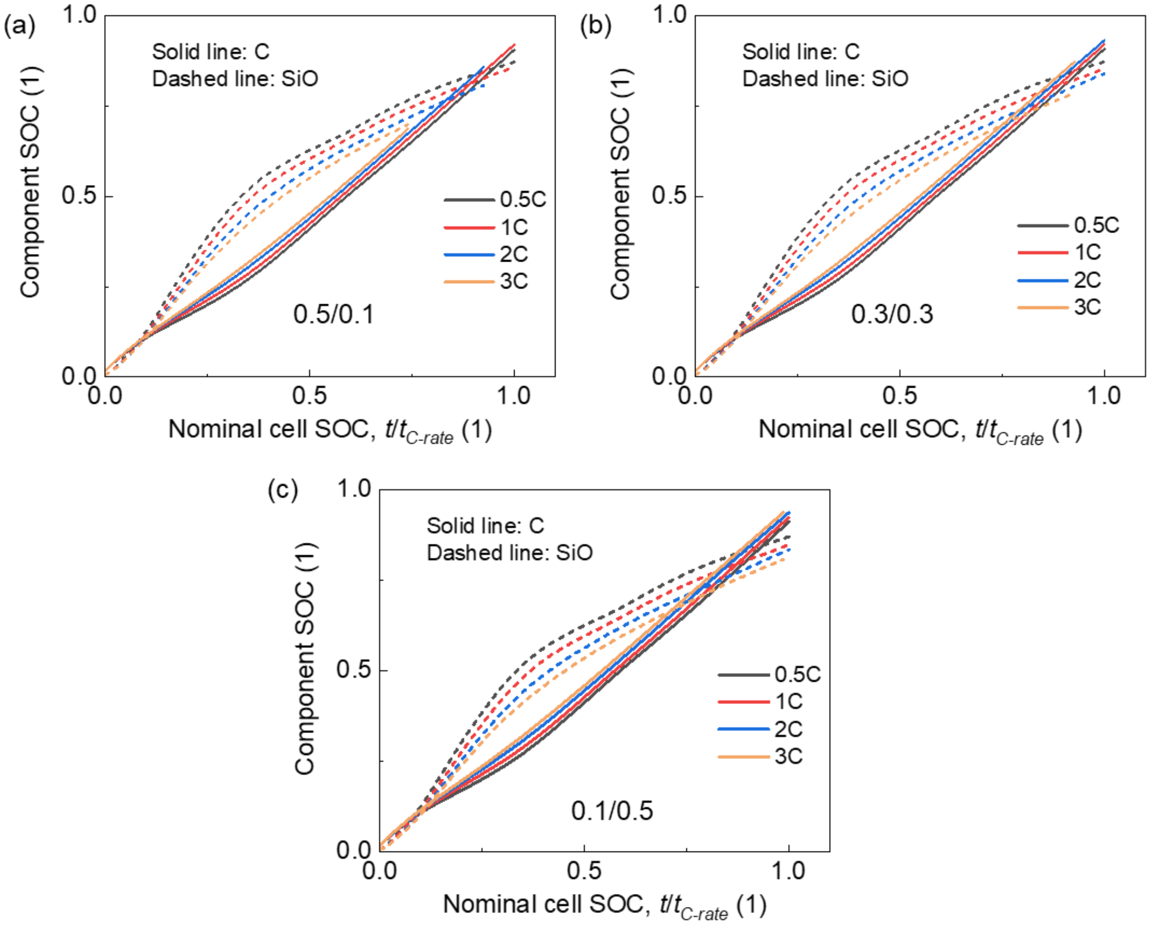

Figure S12. Component SOC profiles under various C rates for the case with CBD inhomogeneity of (a) 0.5/0.1, (b) 0.3/0.3, and (c) 0.1/0.5.

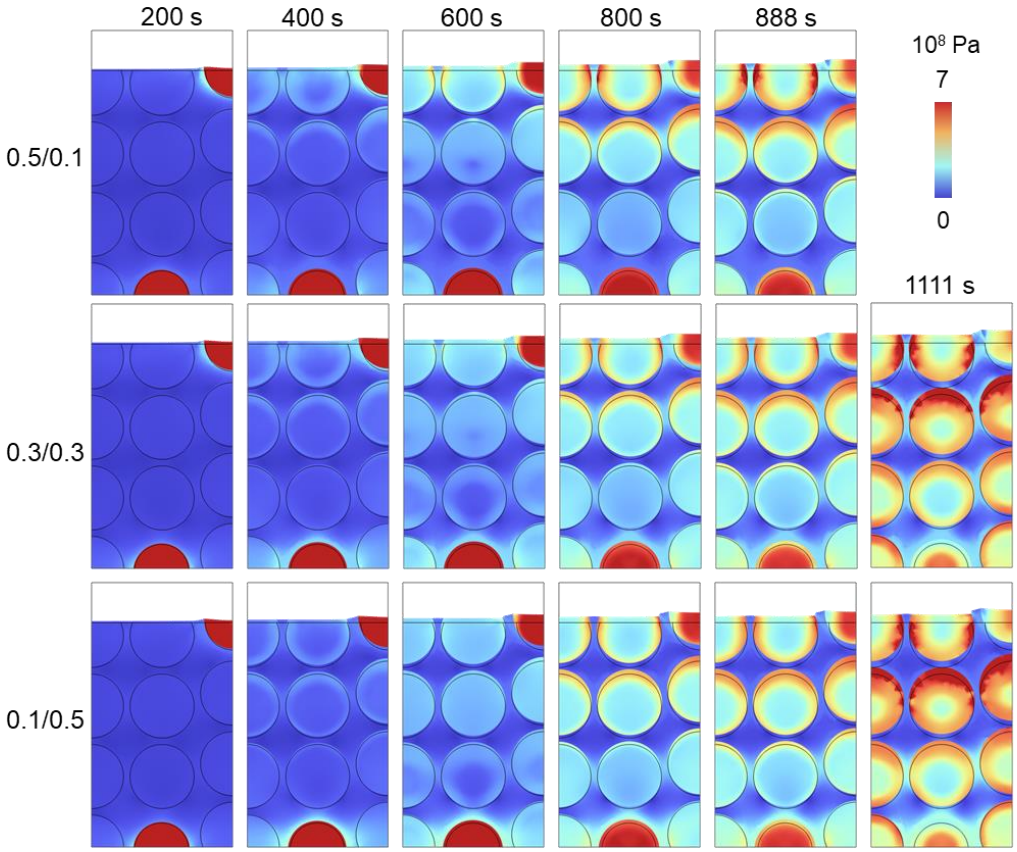

Figure S13. Stress evolution during the lithiation at five selected states (six states for cases-0.3/0.3 and 0.1/0.5) in anodes with three representative CBD inhomogeneities under 3C rate condition.

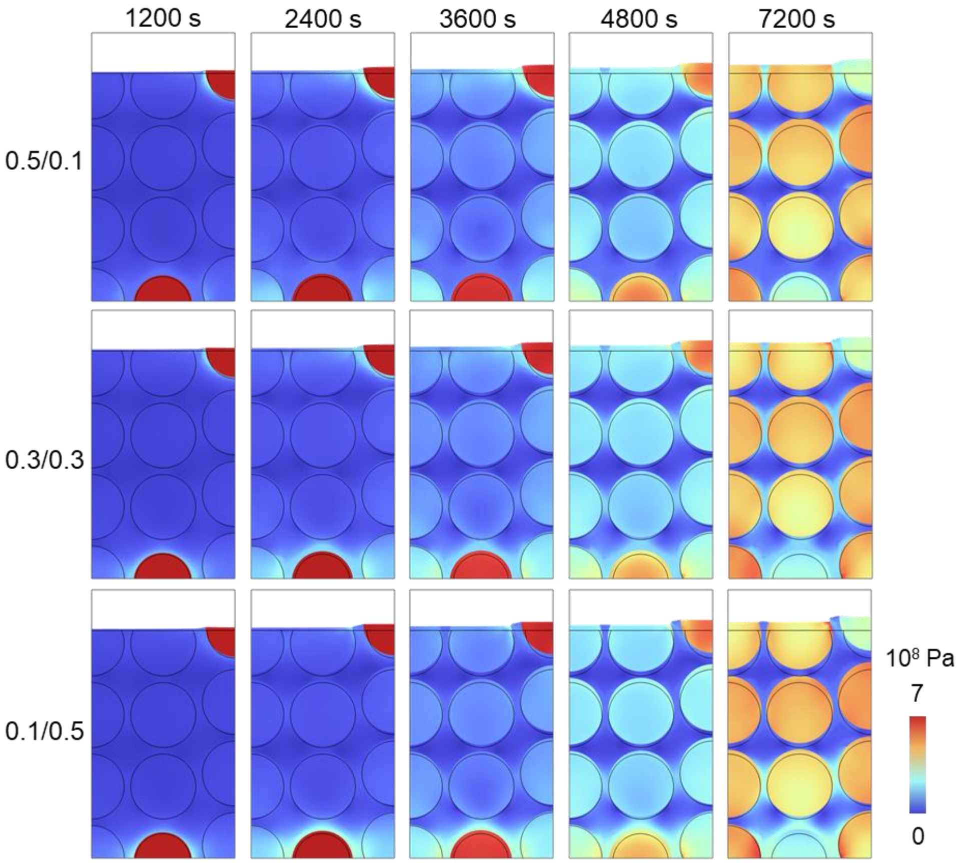

Figure S14. Stress evolution during the lithiation at five selected states in anodes with three representative CBD inhomogeneities under 0.5C rate condition.

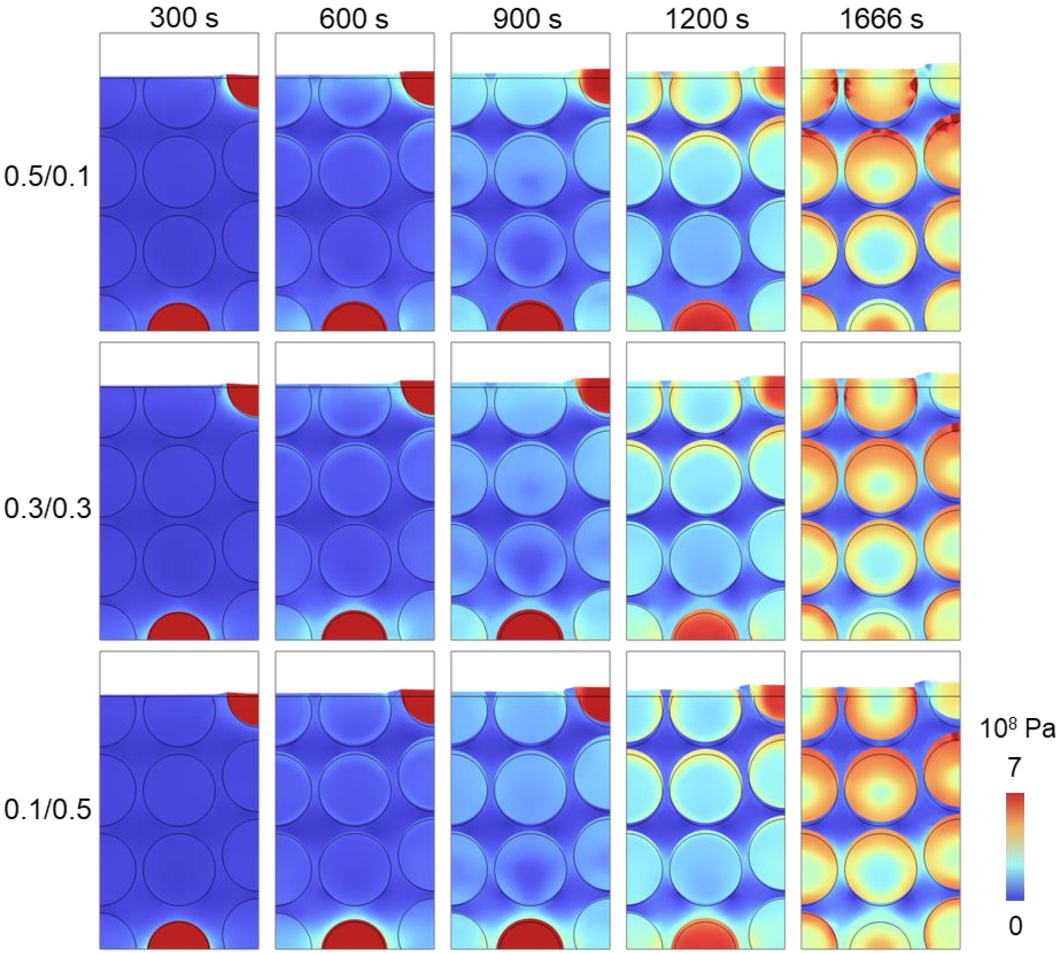

Figure S15. Stress evolution during the lithiation at five selected states in anodes with three representative CBD inhomogeneities under 2C rate condition.

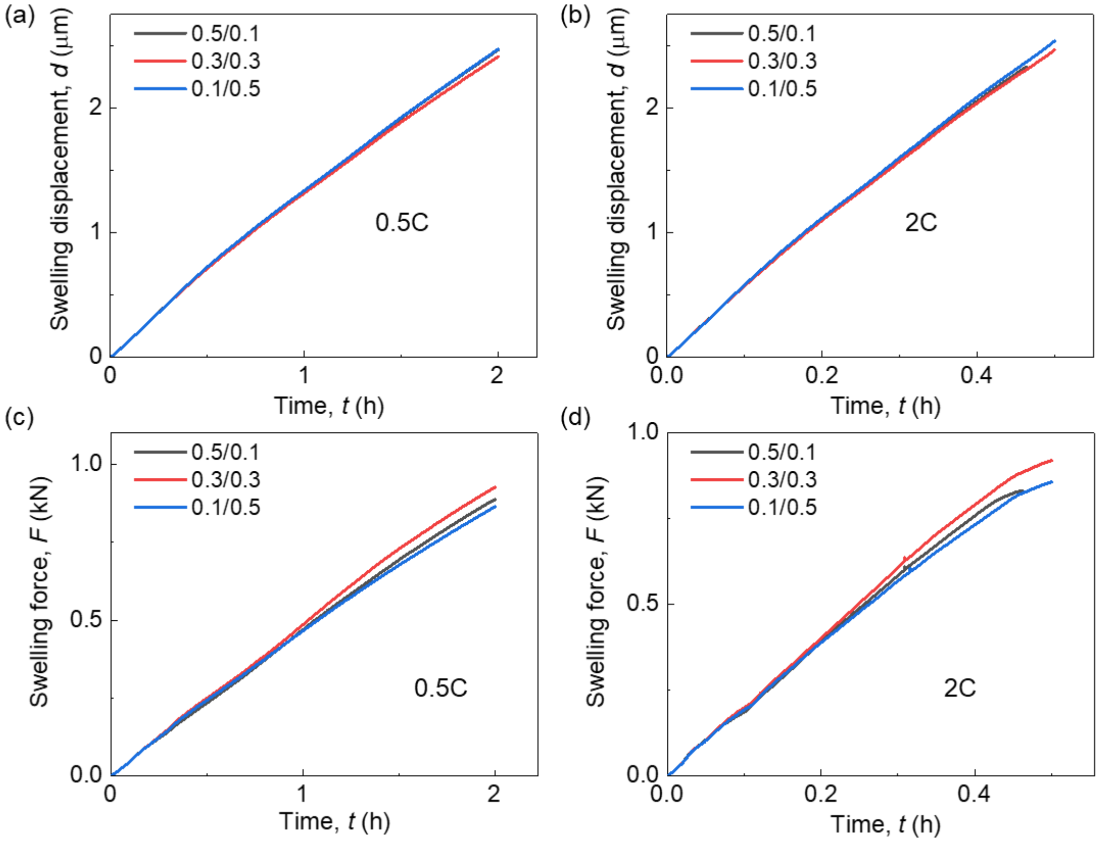

Figure S16. Electrode averaged lithiation induced deformation under (a) 0.5C rate condition and (b) 2C rate condition, and the averaged in-plane force under (c) 0.5C rate condition and (d) 2C rate condition in anodes with three representative CBD inhomogeneities.

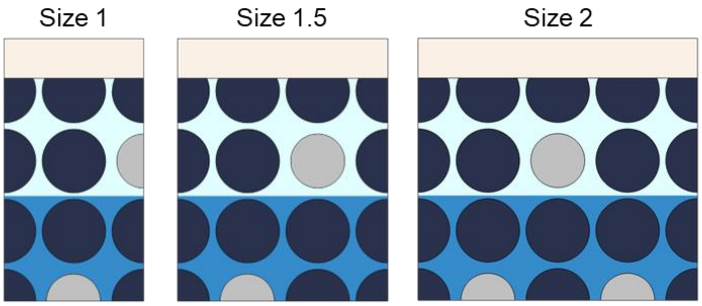

Figure S17. The model configurations for the study on RVE size convergence are as follows: Size 1 corresponds to the dimensions used throughout the entire paper. Size 1.5 has a width that is 1.5 times greater than that of Size 1, and Size 2 has a width that is twice as large as that of Size 1.

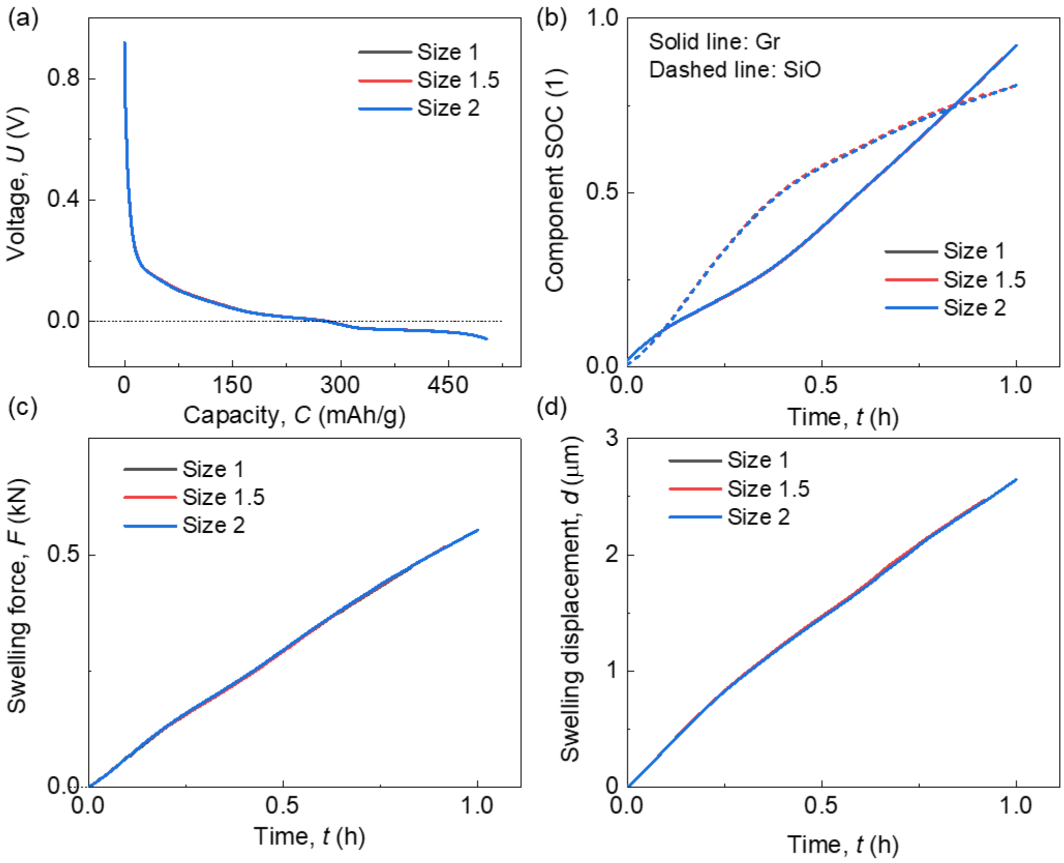

Figure S18 The results comparison among the three RVE size models in terms of (a) half-cell voltage profiles; (b) component SOC profiles; (c) swelling force curves; (d) swelling displacement curves.

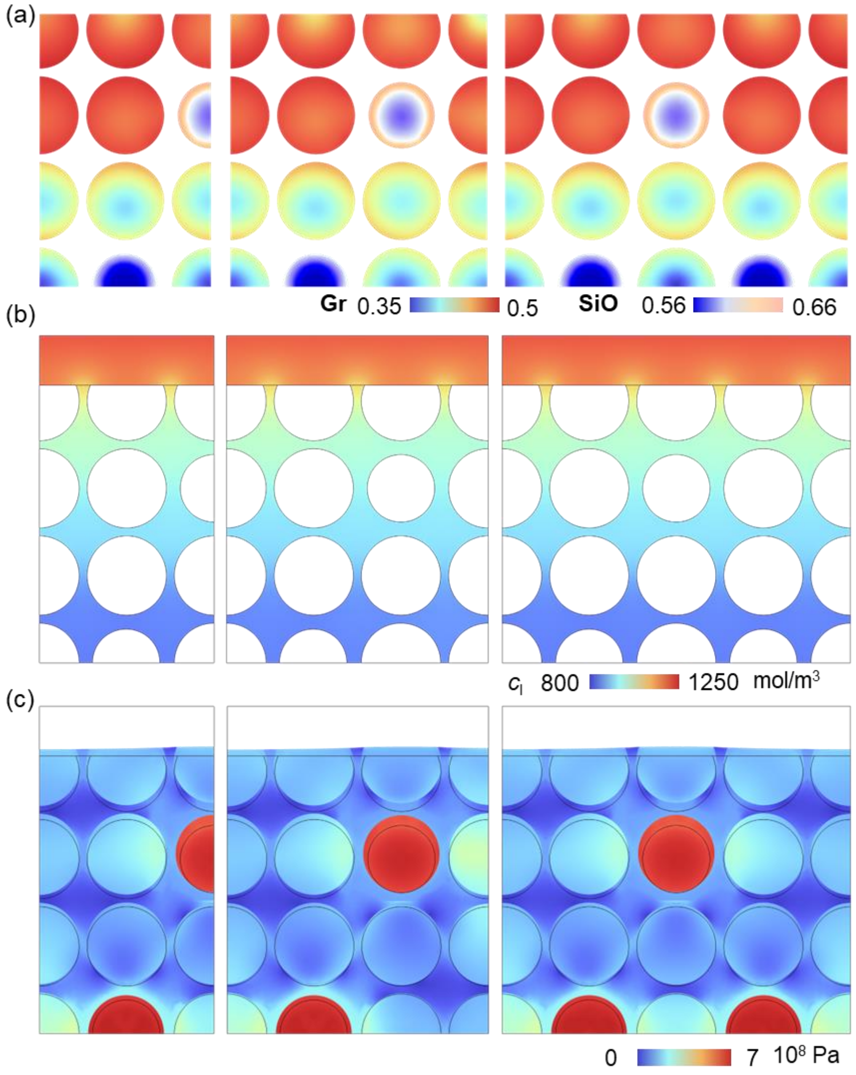

Figure S19 The comparison of results among the three RVE size models based on the contour plots at a selected representative time point (~2000s) of: (a) component SOCs; (b) Li^+ concentration in the electrolyte; (c) deformation and Von Mises stress.

Supplementary Note 1

The CBD component exists in the form of a porous open foam structure within the electrode, and its framework is established through a combination of additive material (typically carbon black) and binder. For the anode and cathode, common binders include

Styrene-Butadiene Rubber (SBR) and Polyvinylidene fluoride (PVDF), respectively. Given that CBD+E is regarded as a homogenized entity, an equivalent material property is assigned to it. The proportion of CBD profoundly influences the porosity of the CBD+E domain.

Drawing upon the mechanical characteristics of porous structures [1-3], an elastic-plastic model has been formulated with a direct correlation to the CBD proportion (depicted in Fig. S20). The CBD+E domain across diverse CBD proportions uniformly shares the same Young's modulus (E_1=2 GPa), while the yield point varies. Notably, the tangent modulus remains consistent (E_2=100 MPa) across all cases featuring varying CBD proportions.

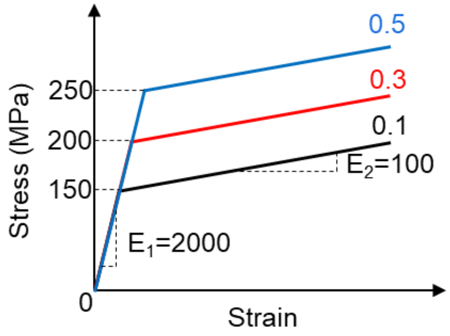

Figure S20. Illustration of the homogenized material model of CBD+E domain.

References:

1. Bekoz, N. and E. Oktay, *Mechanical properties of low alloy steel foams: Dependency on porosity and pore size.* Materials Science and Engineering: A, 2013. **576**: p. 82-90.
2. Zhu, Y., et al., *Numerical simulation of static mechanical properties of PMMA microcellular foams.* Composites Science and Technology, 2020. **192**: p. 108110.
3. Chen, Q., et al., *Modelling of the strength–porosity relationship in glass-ceramic*

foam scaffolds for bone repair. Journal of the European Ceramic Society, 2014. **34**(11): p. 2663-2673.
